# Arboreal snail genus *Amphidromus* Albers, 1850 of Southeast Asia: Shell polymorphism of *Amphidromus cruentatus* (Morelet, 1875) revealed by phylogenetic and morphometric analyses

**DOI:** 10.1371/journal.pone.0272966

**Published:** 2022-08-29

**Authors:** Chi-Tse Lee, Chih-Wei Huang, Chung-Chi Hwang, Chirasak Sutcharit, Parin Jirapatrasilp

**Affiliations:** 1 Department of Life Sciences, National Chung Hsing University, Taichung, Taiwan; 2 School of Life Science, National Taiwan Normal University, Taipei, Taiwan; 3 Department of Life Sciences, National University of Kaohsiung, Kaohsiung, Taiwan; 4 Animal Systematics Research Unit, Department of Biology, Faculty of Science, Chulalongkorn University, Bangkok, Thailand; Universiti Malaysia Sabah, MALAYSIA

## Abstract

Species of colourful arboreal snails of the genus *Amphidromus* from Southeast Asia commonly exhibit high intraspecific variation in shell morphology. Although highly polymorphic *Amphidromus* specimens with different colouration have been collected at the same locality and were revealed to possess similar genital organs, there is yet no morphometric or DNA analyses of these different shell morphs. This study is the first to reveal that both striped and stripeless morphs of *A*. *cruentatus* from Laos and Vietnam belong to the same mitochondrial (COI and 16S rRNA) lineage. Although the shell colouration between the striped and stripeless morphs is markedly different, morphometric and shell outline-based analyses indicated an overall similarity in shell shape. We also revised the systematics of *A*. *cruentatus*, in which we treated similar related species, namely *A*. *eudeli*, *A*. *fuscolabris*, *A*. *thakhekensis*, *A*. *gerberi bolovenensis*, *A*. *goldbergi*, *A*. *pengzhuoani*, *A*. *eichhorsti* and *A*. *pankowskiae* as junior synonyms of *A*. *cruentatus*. *Amphidromus daoae*, *A*. *anhdaoorum*, *A*. *stungtrengensis*, *A*. *yangbayensis* and *A*. *yenlinhae*, which were formerly regarded as junior synonyms, are considered as species different from *A*. *cruentatus* based on shell morphology and morphometric analyses. Preliminary phylogenetic analyses also retrieved some *Amphidromus* species groups as distinct mitochondrial lineages.

## Introduction

Southeast Asia, while facing dramatic biodiversity loss, still harbors an exceptionally high degree of biodiversity and endemism among its terrestrial fauna [1–3]. Land snails are one such group that has begun to gain more attention from local researchers, and its diversity has been recently revealed by molecular phylogenetics [4–6]. The arboreal snail genus *Amphidromus* Albers, 1850 is one of the most diverse groups of colorful tree-dwelling snails, but its molecular systematics has not yet been studied intensively. This genus has a distribution ranging from Southern China in the north, covering most of the Southeast Asian mainland, and is limited by Weber’s Line in the southeast [7], with an exceptional single species reported from Northern Australia [8]. Most *Amphidromus* species are known only from shell characters, and exhibit extremely high intraspecific and interspecific variation of shell colouration [7, 9, 10]. Internal anatomical features have been described for a few species and are regarded as more informative in developing hypotheses on systematic relationships [9–11].

A recent revision of *A*. *fuscolabris* Möllendorff, 1898 from Laos revealed that two distinct shell morphs found in sympatry had identical genitalia [9]. The first morph [9: figs 9e, 13j, k] exhibited a shell closely similar to the holotype [9: fig. 13i]. The second morph was monochrome yellowish in colour [9: figs 9f, 13l, m] and closely similar to *A*. *cruentatus* (Morelet, 1875) [12: fig. 6f]. *Amphidromus eudeli* Ancey, 1897, another species described from Binh Dinh, Annam, and which is similar to *A*. *fuscolabris*, has so far been accepted as a distinct species [7, 9, 13].

In the past decade, several *Amphidromus* species similar to *A*. *cruentatus*, *A*. *eudeli* and *A*. *fuscolabris* have been described from Laos and Vietnam [14–19]. This has led to some arguments both for and against synonymization of these taxa. *Amphidromus thakhekensis* Thach & Huber, 2017 was firstly treated as a synonym of *A*. *fuscolabris* [20]. Later, *A*. *daoae* Thach, 2016 and *A*. *daoae robertabbasi* Thach, 2017 were synonymized with *A*. *cruentatus*; *A*. *yangbayensis* Thach & Huber, 2016 and *A*. *yenlinhae* Thach & Huber, 2017 with *A*. *eudeli*; and *A*. *anhdaoorum* Thach, 2017, *A*. *goldbergi* Thach & Huber, 2018, *A*. *pengzhuoani* Thach, 2018, and *A*. *stungtrengensis* Thach & Huber, 2018 with *A*. *fuscolabris* [13]. However, some arguments against these synonymizations were provided in defense of the validity of these recently described taxa [18, 21, 22].

The conflict in treating the status of those *Amphidromus* taxa either as synonyms or valid species has occurred because *Amphidromus* commonly exhibits intraspecific shell variability [9–11]. This conflict is also caused by the adoption of different species concepts; some authors followed the typological species concept [23] and examined only shell-based morphology, while disregarding other lines of evidence and not taking meaningful estimates of intraspecific diversity into account. In order to resolve this conflict, we treat species as scientific hypotheses and test their biological status against well-defined criteria. Therefore, we apply an integrative approach, combining morphometric and molecular phylogenetic analyses to scrutinize the taxonomic status of *A*. *cruentatus*, *A*. *eudeli*, *A*. *fuscolabris* and those conchologically similar nominal species.

## Materials and methods

### Specimen preparation

**Fig 1:**
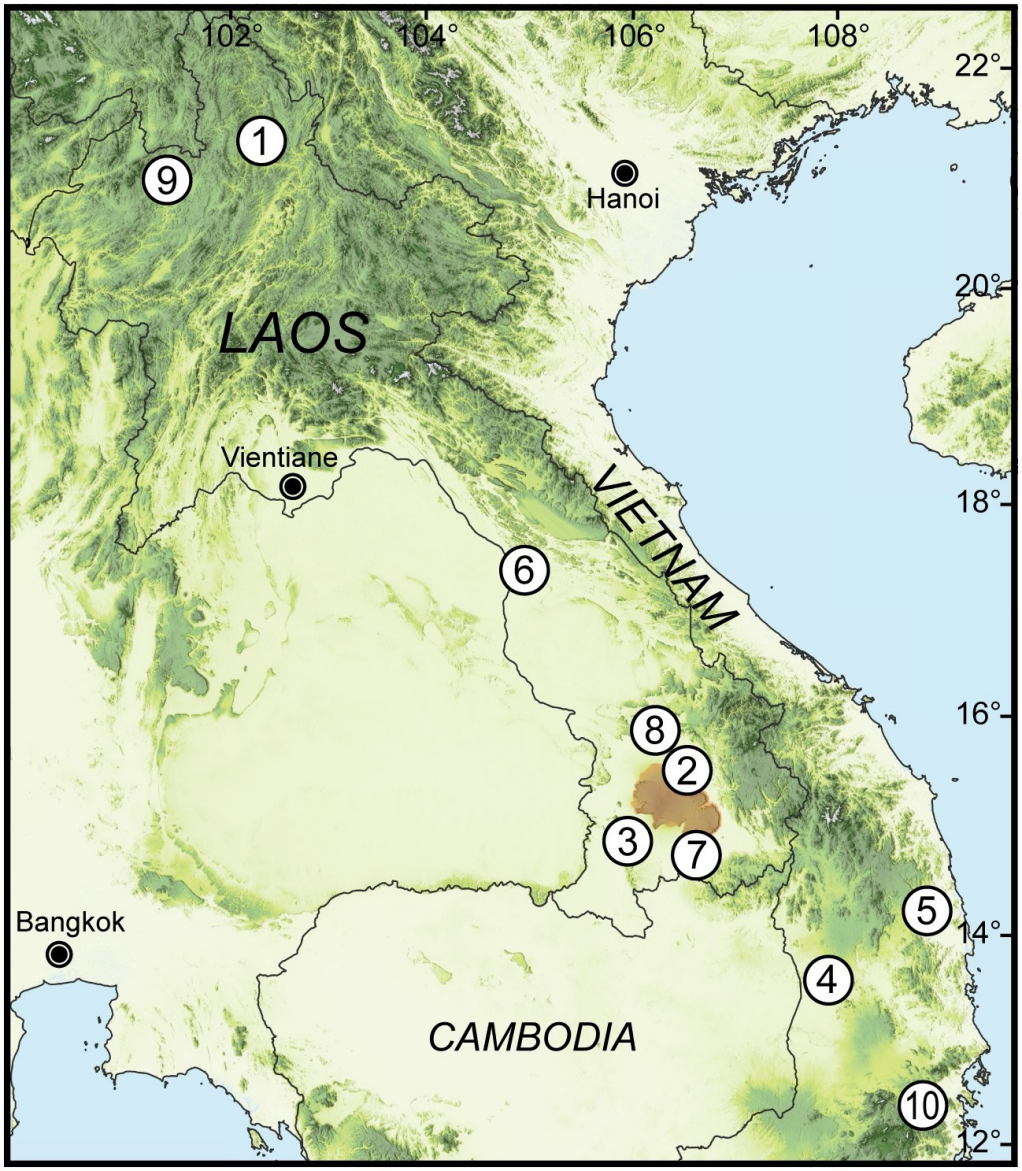
Occurrence records of *Amphidromus cruentatus* examined in this study. No. 1: Samphanh, Phongsali, Laos; 2: Ban Phone, La-Marm, Sekong, Laos; 3: Ban Xai Na Pho, Phatumphone, Champasak, Laos; 4: Chu Prong, Gia Lai, Vietnam; 5: Binh Dinh, Vietnam, type locality of *A*. *eudeli*; 6: Thakhek, Khammouane, Laos, type locality of *A*. *thakhekensis*; 7: Naoh, Boloven Plateau, Attapeu, Laos, type locality of *A*. *gerberi bolovenensis*; 8: Salavan, Laos, type locality of *A*. *goldbergi*; 9: Luang Namtha, Laos, type locality of *A*. *pengzhuoani*; 10: Northwestern District, Khánh Hòa, Vietnam, type locality of *A*. *pankowskiae*. Orange shaded area indicates Boloven Plateau, type locality of *A*. *fuscolabris*. The type localities of *A*. *cruentatus* and *A*. *eichhorsti* (Cambodia and North Laos, respectively) are general and not indicated in the figure. The map was produced using QGIS (3.16.0) with SRTM Downloader plugin (https://github.com/hdus/SRTM-Downloader), retrieving SRTM data from NASA Earth Data server (https://urs.earthdata.nasa.gov/).

**Fig 2:**
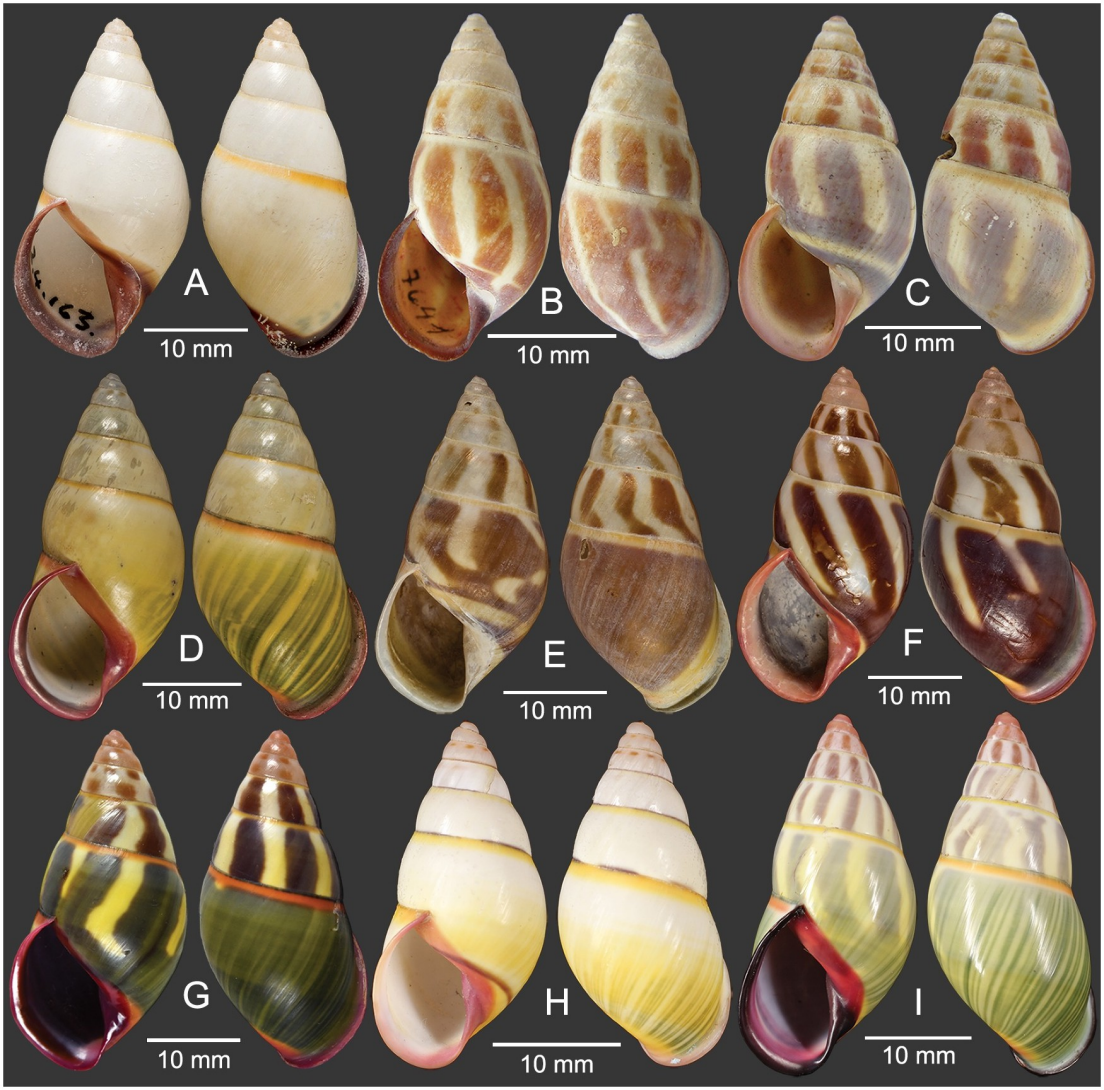
Type specimens of *Amphidromus cruentatus*. A. Holotype of *Bulimus cruentatus*, NHMUK 1893.2.4.163; B. Holotype of *A*. *zebrinus fuscolabris*, SMF 7641; C. Syntype of *A*. *eudeli*, RBINS 617427; D. Holotype of *A*. *thakhekensis*, MNHN-IM-2000-33216; E. Holotype of *A*. *gerberi bolovenensis*, MNHN-IM-2000-34074; F. Holotype of *A*. *goldbergi*, MNHN-IM-2000-34073; G. Holotype of *A*. *pengzhuoani*, NHMUK. 20180243; H. Holotype of *A*. *eichhorsti*, MNHN-IM-2000-35554; and I. Holotype of *A*. *pankowskiae*, MNHN-IM-2000-35543. Credit: V. Héros, P. Maestrati (D–F, H, I).

**Fig 3:**
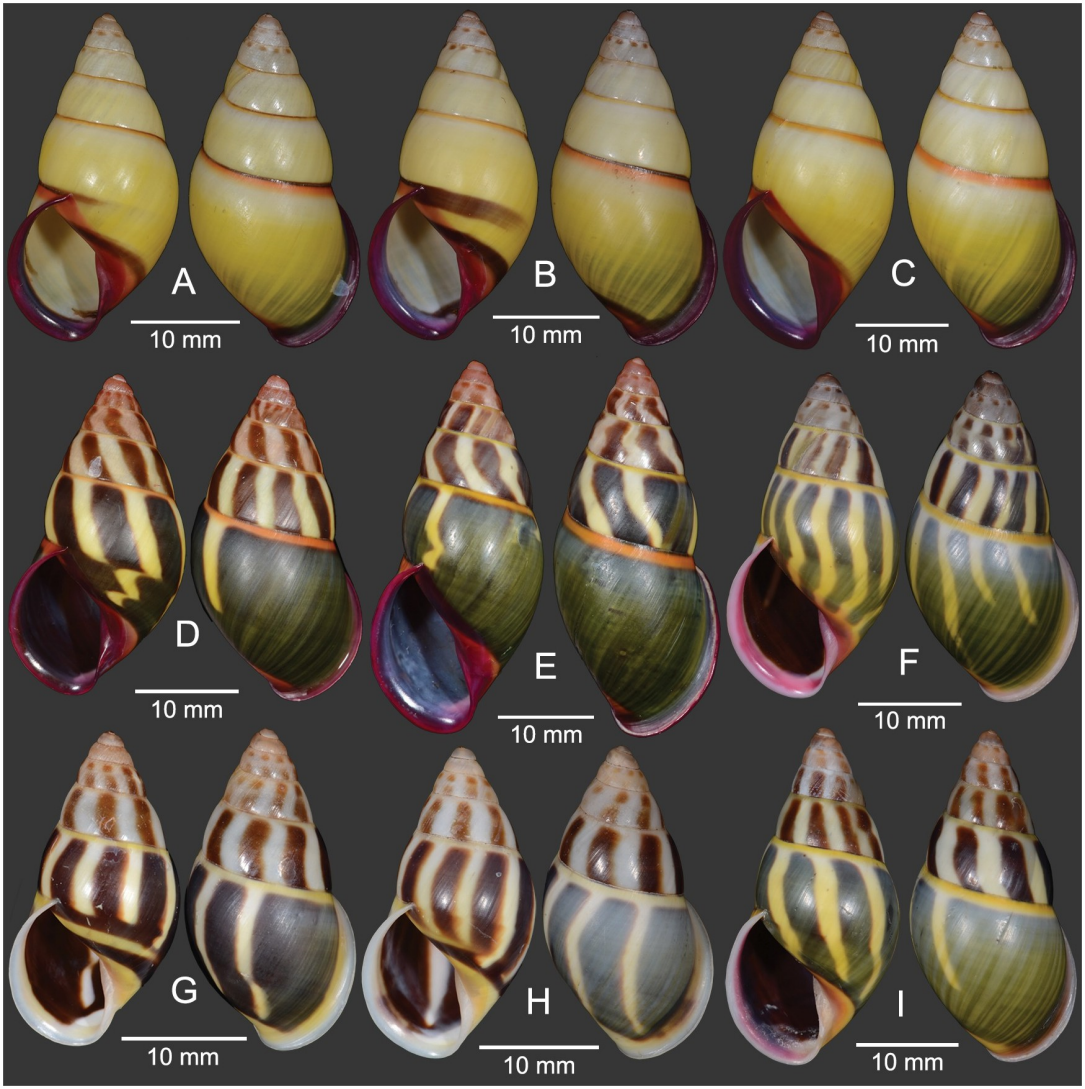
Specimens of *Amphidromus cruentatus*. A–C. Stripeless morph and D–F. striped morph from Samphanh, Phongsali, Laos; G–I. Striped morph from Chu Prong, Gia Lai, Vietnam.

**Fig 4:**
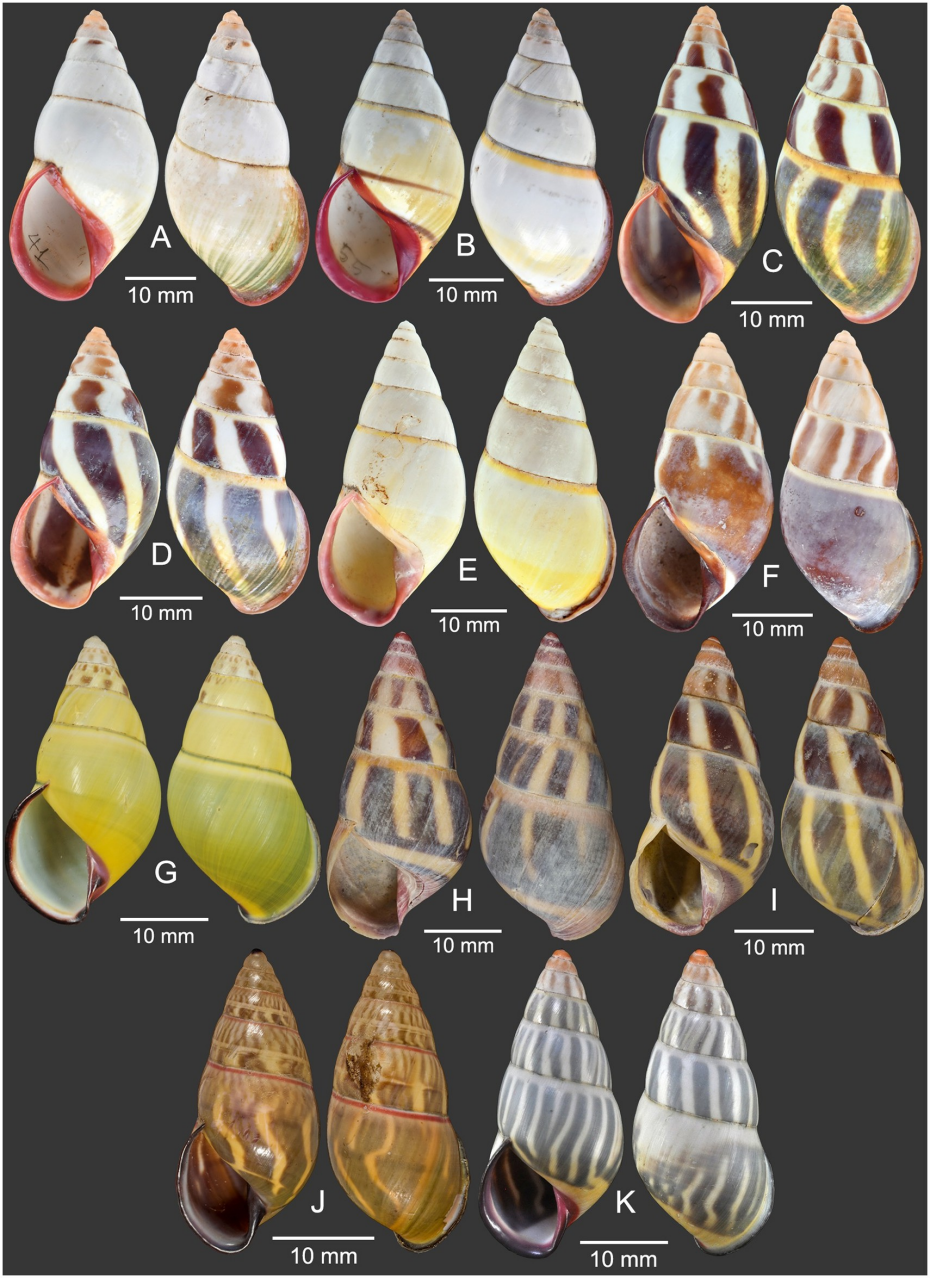
Specimens of *Amphidromus cruentatus* and type specimens of other nominal *Amphidromus* species. A–F. Specimens of *A*. *cruentatus*; A, B. Stripeless morph and C, D. striped morph from Ban Phone, La-Marm, Sekong, Laos; E. Stripeless morph and F. striped morph from Ban Xai Na Pho, Phatumphone, Champasak, Laos; G. Holotype of *A*. *daoae*, RMNH.5004201; H. Holotype of *A*. *yangbayensis*, MNHN-IM-2000-32435; I. Holotype of *A*. *yenlinhae*, MNHN-IM-2000-33230; J. Holotype of *Amphidromus stungtrengensis*, MNHN-IM-2000-34084; and K. Holotype of *A*. *anhdaoorum*, MNHN-IM-2000-33232. Credit: J. Goud (G), V. Héros, P. Maestrati (H–K).

This study is mainly based on shells and preserved specimens from Laos and Vietnam, and type specimens of *A*. *cruentatus* and conchologically similar nominal species (Figs 1–4, Table 1). Three shell morphs of newly collected specimens were discriminated following Inkhavilay *et al*. [9] and Sutcharit *et al*. [12]: (1) the monochrome yellow stripeless morph of “*A*. *cruentatus*” collected from Samphanh, Phongsali, Laos (Figs 2A and 3A–3C), (2) the striped morph of “*A*. *fuscolabris*” collected from the same locality (Figs 2B and 3D–3F), and (3) the striped morph of “*A*. *eudeli*” collected from Chu Prong, Gia Lai, Vietnam (Figs 2C and 3G–3I). At each collecting site, the specimens were collected within an area of approximately 100 m^2^. Additional voucher specimens of *A*. *fuscolabris* from Ban Phone, La-Marm, Sekong (Fig 4A–4D) and Ban Xai Na Pho, Phatumphone, Champasak, Laos (Fig 4E and 4F) from the collection of Chulalongkorn University Museum of Zoology (CUMZ), containing both striped and stripeless morphs [9] were also included in this study. Shells of *A*. *atricallosus* (Gould, 1843) and *A*. *inversus* (Müller, 1774) from Myanmar, Thailand and Malaysia were included for comparison in the morphometric analysis (Table 1).

**Table 1:** List of *Amphidromus* specimens used in morphometric analyses.

Species	Locality	Voucher number	No. of specimen and chirality	Figure
*A*. *cruentatus*	Samphanh, Phongsali, Laos	NMNS-8476-034 to NMNS-8476-051 (stripeless morph)	18S	Fig 3A–3C
		NMNS-8476-001 to NMNS-8476-033 (striped morph)	33S	Fig 3D–3F
	Ban Phone, La-Marm, Sekong, Laos	CUMZ 7042 (stripeless morph)	20S	Fig 4A and 4B
		CUMZ 7040 (striped morph)	20S	Fig 4C and 4D
	Ban Xai Na Pho, Phatumphone, Champasak, Laos	CUMZ 7044/2 (stripeless morph)	1S	Fig 4E
		CUMZ 7044/1 (striped morph)	1S	Fig 4F
	Chu Prong, Gia Lai, Vietnam	NMNS-8476-052 to NMNS-8476-066 (striped morph)	15S	Fig 3G–3I
	Cambodia	Holotype NHMUK 1893.2.4.163	1S	Fig 2A
	near Binh Dinh, Vietnam	Syntype of *A*. *eudeli* Ancey, 1897, RBINS 617427	1S	Fig 2C
	Boloven Plateau, Paksong, Champasak, Laos	Holotype of *A*. *zebrinus fuscolabris* Möllendorff, 1898, SMF 7641	1S	Fig 2B
	Thakhek, Khammouane, Laos	Holotype of *A*. *thakhekensis* Thach & Huber, 2017, MNHN-IM-2000-33216	1S	Fig 2D
	Naoh, Attapeu, Laos	Holotype of *A*. *gerberi bolovenensis* Thach & Huber, 2018, MNHN-IM-2000-34074	1S	Fig 2E
	Salavan, Laos	Holotype of *A*. *goldbergi* Thach & Huber, 2018, MNHN-IM-2000-34073	1S	Fig 2F
	Luang Namtha, Laos	Holotype of *A*. *pengzhuoani* Thach, 2018, NHMUK 20180243	1S	Fig 2G
	North Laos	Holotype of *A*. *eichhorsti* Thach, 2020, MNHN-IM-2000-35554	1S	Fig 2H
	Northwestern District of Khánh Hòa, Vietnam	Holotype of *A*. *pankowskiae* Thach, 2020, MNHN-IM-2000-35543	1S	Fig 2I
*A*. *daoae*	Ea Tu Commune, Banmethuot City, Dak Lak, Vietnam	Holotype RMNH.5004201	1S	Fig 4G
*A*. *yangbayensis*	Yangbay, Khanh Vinh, Vietnam	Holotype MNHN-IM-2000-32435	1S	Fig 4H
*A*. *yenlinhae*	Mangto, North of La Nga River, Binh Thuan, Vietnam	Holotype MNHN-IM-2000-33230	1S	Fig 4I
*A*. *stungtrengensis*	Stung Treng, Cambodia	Holotype MNHN-IM-2000-34084	1S	Fig 4J
*A*. *anhdaoorum*	Krong Bong, Daklak, Vietnam	Holotype MNHN-IM-2000-33232	1S	Fig 4K
*A*. *atricallosus*	Buddha Cave, Lenya, Tanintharyi, Myanmar	CUMZ 5277	6D+3S	-
	Kui Buri, Prachuap Khiri Khan, Thailand	CUMZ 5276	5D+5S	-
	Ban Takhun, Surat Thani, Thailand	CUMZ 5278	3D+3S	-
	Phung Chang Cave, Phang Nga, Thailand	CUMZ 2279	5D+5S	-
*A*. *inversus*	Pulau Kapas, Terengganu, Malaysia	CUMZ 2327	5D+5S	-
	Na Muang Waterfall, Samui Island, Surat Thani, Thailand	CUMZ 5275	5D	-
	Phai Island, Chon Buri, Thailand	CUMZ 2037	10D	-
	Elar Island, Chon Buri, Thailand	CUMZ 2229	10D	-
	Kaeng Lamduan, Ubon Ratchathani, Thailand	CUMZ 5274	3D+2S	-

Living specimens were placed in a -20°C freezer and subsequently transferred to 95% ethanol following the two-step method for euthanasia [24]. All specimens were compared with the relevant type specimens. The type locality provided is from the original publication in the original wording and language. If possible, the modern name and/or regional names of the type locality are provided in square brackets. The newly obtained specimens were deposited as vouchers in the National Museum of Natural Science of Taiwan (NMNS).

### Molecular phylogenetic analysis

Mitochondrial COI and 16S sequences were obtained from eight specimens of stripeless “*A*. *cruentatus*” and nine specimens of striped “*A*. *fuscolabris*” from Samphanh, Laos, and three specimens of striped “*A*. *eudeli*” from Chu Prong, Vietnam (S1 Table). Genomic DNA was extracted from foot tissue of snails using CTAB method [25]. Fragments of mitochondrial cytochrome *c* oxidase subunit 1 (CO1) and large ribosomal RNA (16S) were amplified using universal primers LCO1490 and HCO2198 for COI [26] and 16Sar and 16Sbr for 16S [27]. The PCR protocol followed Huang *et al*. [28]. PCR products were checked via gel electrophoresis and sequenced by Genomics or Mission Biotech (Taiwan). Sequences were checked manually using BioEdit version 7.2.6 [29], and primer sequences were trimmed before alignment. We incorporated DNA sequences of other *Amphidromus* species available in GenBank [30–33] into the dataset (S1 Table). Sequences of *Camaena cicatricosa* (Müller, 1774) (type species of *Camaena*), *C*. *poyuensis* Zhou, Wang & Ding, 2016 and *Trichelix hiraseana* (Pilsbry, 1905) (Camaeninae), and *Cornu aspersum* (Müller, 1774) (Helicidae) were also retrieved [34, 35] and used as outgroup.

Multiple sequence alignment was conducted using ClustalW [36] implemented in BioEdit. Maximum likelihood (ML) phylogeny of the concatenated dataset was reconstructed using IQTREE webserver (http://iqtree.cibiv.univie.ac.at) with integrated ModelFinder function [37–39]. One hundred thousand replicates of ultrafast bootstrap approximation were conducted using UFBoot [40], and the Shimodaira and Hasegawa-approximate likelihood-ratio (SH-aLRT) test and the approximate Bayes (aBayes) test [41] were also conducted in order to assess the support for nodes of phylogeny. Kakusan4 [42] was implemented to prepare the concatenated dataset with the best-fitting model adjustment for Bayesian inference (BI) analyses. The BI analysis was performed with the best-fitting models of each gene fragment and each codon position of COI using MrBayes on XSEDE v.3.2.6 [43] in the CIPRES Science Gateway [44]. Two independent analyses were run simultaneously, each consisting of four chains of 10 million generations. The sampling rate was 500 generations, and the first 50% of sampled trees were discarded as burn-in. A clade was considered to be well supported if the ultra-fast bootstrap support (BS) values were ≥ 95%, aBayes support values ≥ 0.95, SH-aLRT support values ≥ 80% and Bayesian posterior probability values (PP) were ≥ 0.95 [40, 41, 45]. The resulting phylogeny was visualized in FigTree version 1.4.4 [46].

Both COI and 16S haplotypes were identified by using DnaSP version 6.12.01 [47]. A median-joining network [48] was generated and visualized using POPART version 1.7 [49]. The genetic distances within the same morph and pairwise distances among different morphs within the same and between different collecting sites, along with intra- and interspecific distances were calculated using *p*-distance by MEGA X [50].

### Morphological analyses

**Fig 5:**
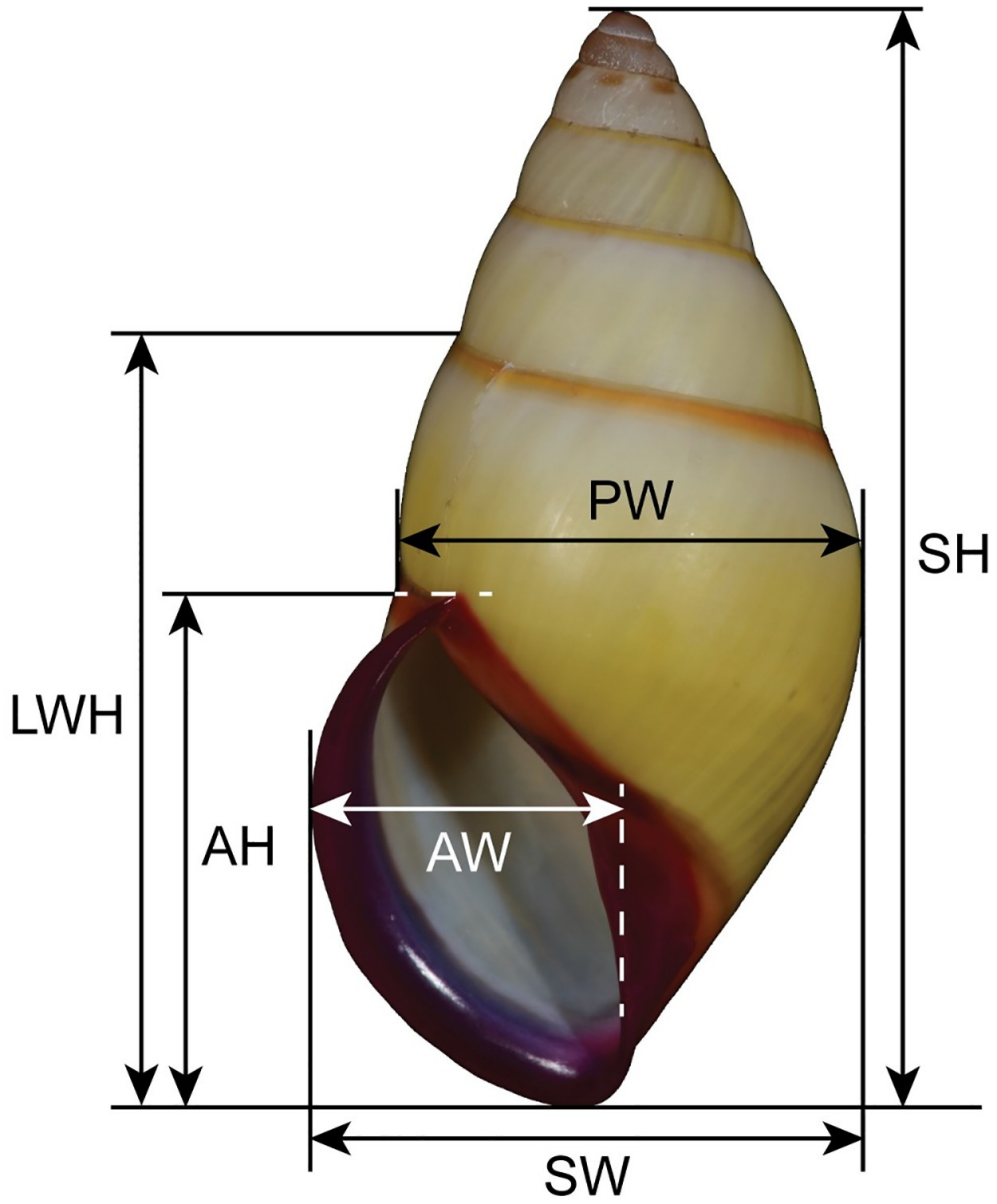
Shell dimensions of *Amphidromus* used in this study. Shell height (H), last whorl height (LWH), shell width (D), penultimate whorl width (PW), apertural height (AH), and apertural width (AW).

Shell and genitalia photographs were taken by Nikon D850 digital single-lens reflex camera. Shell banding patterns were described following Inkhavilay *et al*. [9] and Wu *et al*. [34], and genital morphology was examined following Inkhavilay *et al*. [9]. Shell dimensions including shell height (H), last whorl height (LWH), shell width (D), penultimate whorl width (PW), apertural height (AH), and apertural width (AW) (Fig 5) were measured in mm by digital Vernier caliper (Mitutoyo, CD-6 CS). The shell height other than last whorl (H-LWH) was calculated via shell height minus last whorl height, and spire height (SpH) was calculated via shell height minus apertural height. The ratios of shell height to shell width (H/D), apertural height to apertural width (AH/AW), shell height to apertural height (H/AH), shell width to apertural width (D/AW), shell height to last whorl height (H/LWH), last whorl height to shell height other than last whorl (LWH/(H-LWH)), last whorl height to apertural height (LWH/AH), spire height to apertural height (SpH/AH), shell width to penultimate whorl width (D/PW), and penultimate whorl width to apertural width (PW/AW) were calculated. Number of whorls was counted to the nearest 0.25 (1/4 whorl) following Haniel [10].

We used Welch’s *t*-test in PAST version 4.04 [51] to analyze the variation in shell parameters between different morphs within the same collecting site and compared among different collecting sites regardless of morph membership. The sequential Bonferroni correction was applied to adjust for multiple test comparisons. Measurements of all shells, including types, were analyzed using principal component analysis (PCA) in Clustvis web tool [52], applying unit variance scaling and singular value decomposition (SVD) with imputation. 0.95-probability prediction ellipses were determined for each morph of *A*. *cruentatus* separately and all morphs combined. For the shell outline-based analysis, specimen photographs were converted to thin plate spline (TPS) format via tpsUtil version 1.79 [53]. The shell shape outline was digitalized using tpsDig version 2.31 [54], and the mean shell shape of each morph was calculated via Morphomatica version 1.6 [55]. Specimens from Ban Xai Na Pho and all type specimens were not included in the Welch’s *t*-test and shell outline-based analyses due to a small sample size of fewer than 10 individuals.

## Results

### Molecular phylogenetic analysis

The COI dataset comprised 29 sequences with lengths between 563 and 642 bp, including 265 variable and 236 parsimony-informative sites, from an alignment length of 642 bp. The 16S rRNA dataset comprised 63 sequences with lengths between 350 and 388 bp. The 16S rRNA alignment including gaps was 403 bp, including 173 variable and 153 parsimony-informative sites.

**Fig 6:**
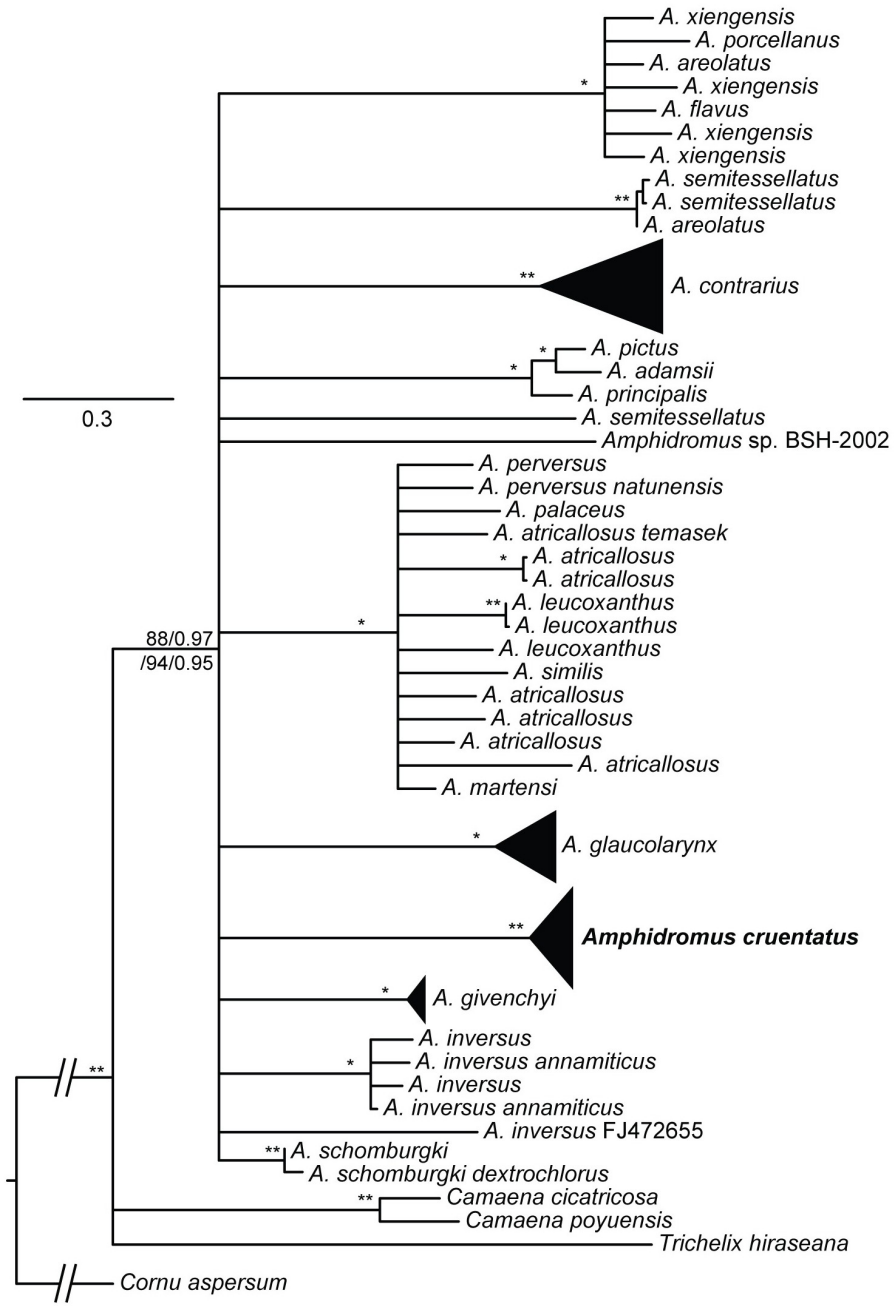
Phylogenetic tree based on maximum likelihood analysis. Nodal support values are given as SH-aLRT/aBayes/ultra-fast bootstrap (IQ-TREE, ML)/posterior probability (MrBayes, BI). Two asterisks on the branch indicate a clade with all well-supported values (SH-aLRT ≥ 80%, aBayes ≥ 0.95, BS ≥ 95%, PP ≥ 0.95), whereas one asterisk indicates a clade well supported by ML but not by BI.

The best-fitting models of each gene fragment and each codon position of COI for Bayesian phylogram construction are as follows: GTR+G for the first, F81+G for the second, and HKY+G for the third codon position of COI and 16S rRNA. The ML and BI phylogenetic analyses based on the concatenated datasets yielded consistent topologies (Fig 6, showing ML topology). However, some clades in the Bayesian phylogram received insufficient statistical support by means of Bayesian posterior clade probabilities. All specimens of “*A*. *cruentatus*”, “*A*. *fuscolabris*”, and “*A*. *eudeli*” were retrieved together in the same clade which was well-supported by all support values, while no taxa were retrieved as monophyletic. Therefore, we regard all specimens of “*A*. *fuscolabris*” and “*A*. *eudeli*” in this study as the striped morph of the oldest valid taxon, *A*. *cruentatus*.

The overall relationships among *Amphidromus* species yielded an unresolved polytomy, although some *Amphidromus* species belong to the same clade. For example, *A*. *pictus*, *A*. *adamsii* and *A*. *principalis* belong to the same clade, while *A*. *atricallosus*, *A*. *leucoxanthus*, *A*. *palaceus*, *A*. *perversus*, *A*. *martensi* and *A*. *similis* belong to another clade (Fig 6). The percentages of uncorrected intraspecific and pairwise interspecific *p*-distances for 16S rRNA ranged between 0.77 and 10.92% (average 4.27 ± 3.35%) and between 4.06 and 19.58% (average 14.70 ± 3.72%), respectively (S2 Table). The average of pairwise interspecific *p*-distances for 16S rRNA between *A*. *cruentatus* and other *Amphidromus* species was 16.51 ± 1.25%.

**Fig 7:**
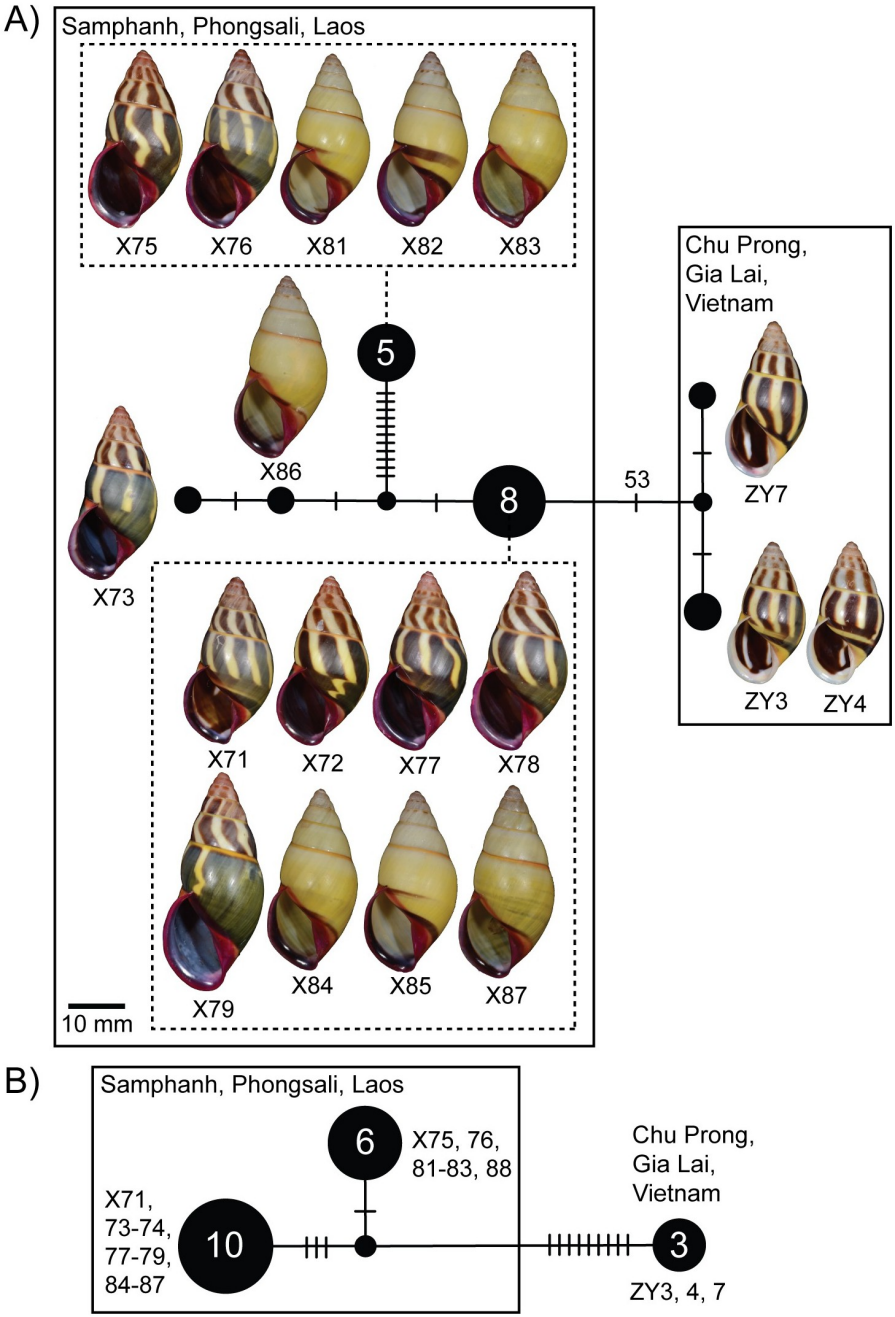
Mitochondrial haplotype minimum spanning networks of *Amphidromus cruentatus*. A. COI and B. 16S rRNA. The size of each circle corresponds to the frequency of that haplotype, also shown as the number in that circle. The cross bars on the branches indicate the number of transitions between haplotypes. Specimen codes correspond to those in Table 1.

There was a total of six COI haplotypes and three 16S haplotypes of *A*. *cruentatus*, and the haplotypes from Samphanh were separated from those from Chu Prong, Gia Lai, Vietnam by 53 and 9 mutations for COI and 16S, respectively, in the minimum spanning network (Fig 7). Genetic distances between striped and stripeless specimens from the same location were lower than the distances between Lao and Vietnamese specimens regardless of morph membership (Table 2). Genetic *p*-distances within the Lao specimens were 0.96% and 0.52% for COI and 16S, respectively, and pairwise genetic *p*-distances between Lao and Vietnamese specimens were 8.52% and 2.91% for COI and 16S, respectively.

**Table 2:** Percentage of pairwise *p*-distances among different morphs of *Amphidromus cruentatus* within the same and between different collecting sites for partial COI (above the diagonal) and 16S rRNA (below the diagonal) gene fragments. Genetic distances within the same morph for COI/16S are shown on the diagonal.

Morph	1.	2.	3.
1. Stripeless morph from Samphanh	1.11/0.59	0.93	8.52
2. Striped morph from Samphanh	0.52	0.90/0.44	8.51
3. Striped morph from Chu Prong	2.84	2.97	0.21/0.00

### Morphological analysis

The results from Welch’s *t*-test revealed that the two morphs of *A*. *cruentatus* found at the same sites did not differ from each other in most shell parameters with statistical significance. In contrast, shells from different sites differed significantly from each other in some shell parameters regardless of morph membership (Table 3, S3 Table).

**Table 3:** Welch’s *t*-test between different morphs of *Amphidromus cruentatus* within the same collecting site and between different collecting sites regardless of morph membership.

Shell parameters	Comparison within the same collecting site		Comparison among different collecting sites regardless of morph membership		
	between stripeless and striped morphs from Samphanh	between stripeless and striped morphs from Ban Phone	between Samphanh and Ban Phone	between Chu Prong and Samphanh	between Chu Prong and Ban Phone
H	1.482	0.032	8.755***	6.016***	12.424***
D	0.414	0.510	9.299***	5.909***	11.684***
AH	0.887	0.703	10.663***	6.064***	13.310***
AW	0.250	0.529	5.071***	4.596***	7.757***
LWH	1.460	0.024	9.730***	6.808***	14.361***
PW	1.578	0.215	8.485***	7.055***	12.787***
H-LWH	1.324	0.040	6.385***	4.341***	8.686***
SpH	1.749	0.462	6.560***	5.406***	10.347***
H/D	2.305	0.876	2.824**	2.882	5.411***
AH/AW	2.571	0.203	13.831***	4.574***	14.414***
H/AH	1.802	1.211	1.636	0.197	0.888
D/AW	1.298	0.190	8.669***	2.991**	10.770***
H/LWH	0.550	0.111	0.242	0.396	0.267
LWH/(H-LWH)	0.495	0.339	0.118	0.485	0.420
LWH/AH	2.194	1.727	2.373	0.018	1.765
SpH/AH	1.802	1.235	1.636	0.197	0.888
D/PW	2.037	1.940	3.820***	0.906	3.406**
PW/AW	3.345**	1.420	3.823***	1.083	3.481**
critical *t* value (*p* = 0.05)	2.0096	2.0244	1.987	1.9977	2.0057

*p < 0.05,

**p < 0.01,

***p < 0.001

(*p*-value adjusted after sequential Bonferroni correction)

**Fig 8:**
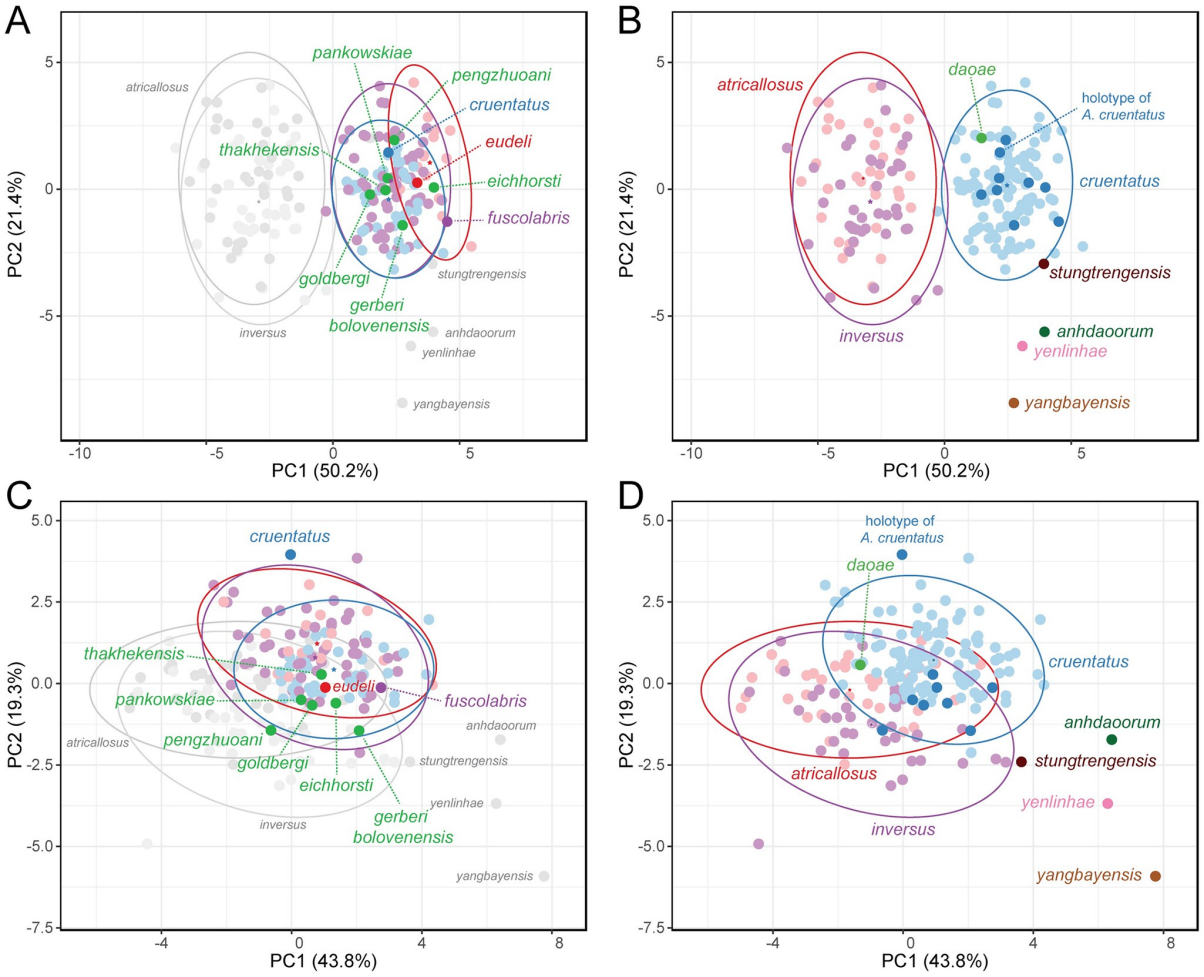
Principle component analyses of shell parameters. A, B. All shell parameters, and C, D. only shell ratios, with separate ellipses (A, C) for each *A*. *cruentatus* morph, and one ellipse (B, D) for all *A*. *cruentatus* specimens with dark blue circles indicating data points of type specimens of nominal species synonymized under *A*. *cruentatus*. Prediction ellipses are based on probability of 0.95 with star as centroid of each ellipse (*N* = 197 data points).

The PCA of all shell parameters (Fig 8A and 8B) identified PC1 and PC2, which explained 50.2% and 21.4% of the total variance, respectively. The three highest loadings of shell parameter variables accounting for PC1 are shell width (0.329), penultimate whorl width (0.326) and apertural width (0.326). The three highest loadings accounting for PC2 are the ratios of shell height to last whorl height (0.413), last whorl height to shell height other than last whorl (0.398), spire height to apertural height and shell height to apertural height (having the same third highest score 0.396). The PCA of the shell ratios only (Fig 8C and 8D) identified PC1 and PC2, which explained 43.8% and 19.3% of the total variance, respectively. The three highest loadings of shell ratio accounting for PC1 are the ratios of spire height to apertural height (0.450), shell height to apertural height (0.450) and shell height to last whorl height (0.413), while the three highest loadings accounting for PC2 are the ratios of penultimate whorl width to apertural width (0.665), shell width to apertural width (0.631) and shell height to shell width (0.294).

**Fig 9:**
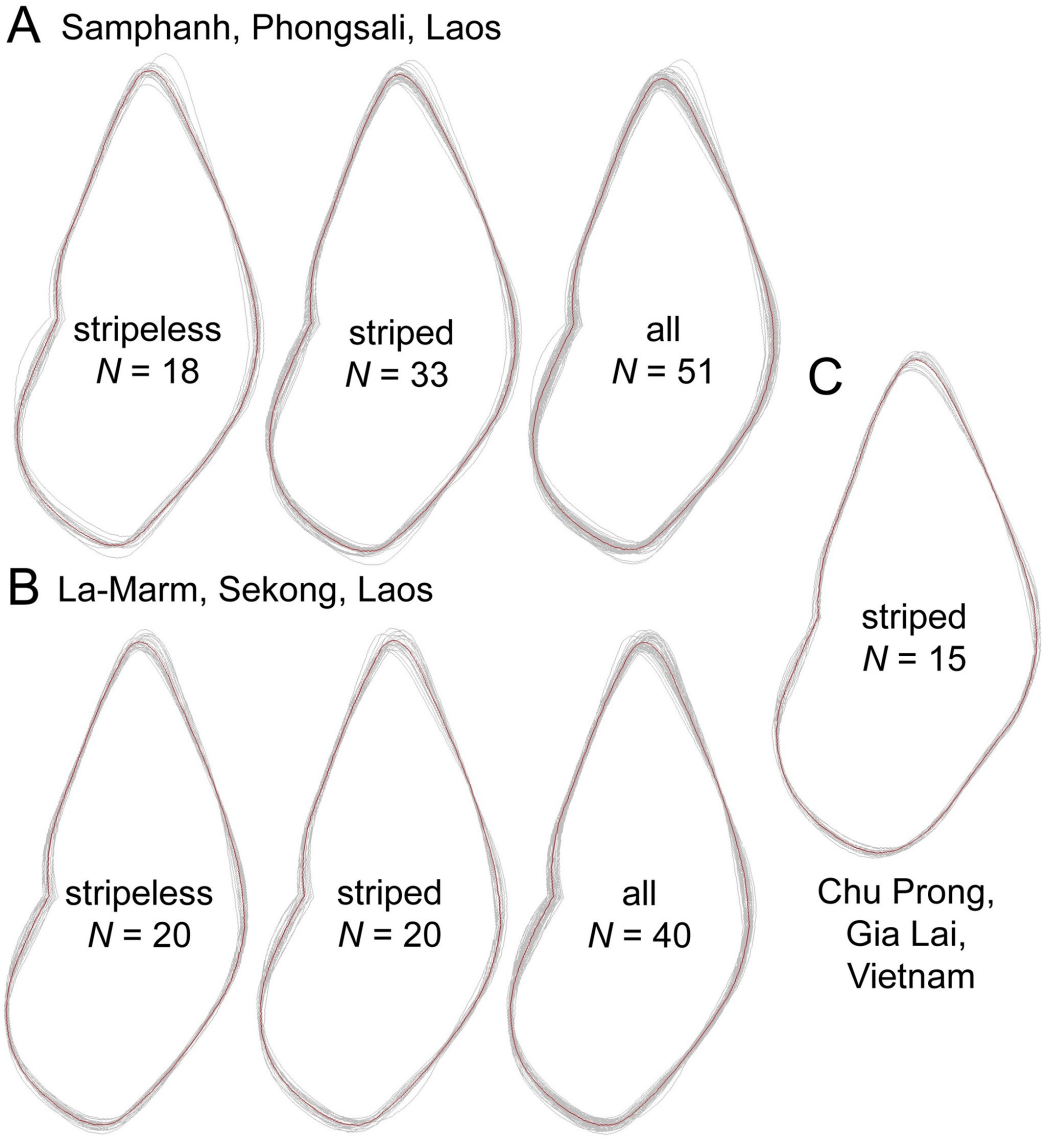
Mean shell shape of *Amphidromus cruentatus* specimens. A. Samphanh, Laos B. Ban Phone, Laos and C. Chu Prong, Vietnam.

**Fig 10:**
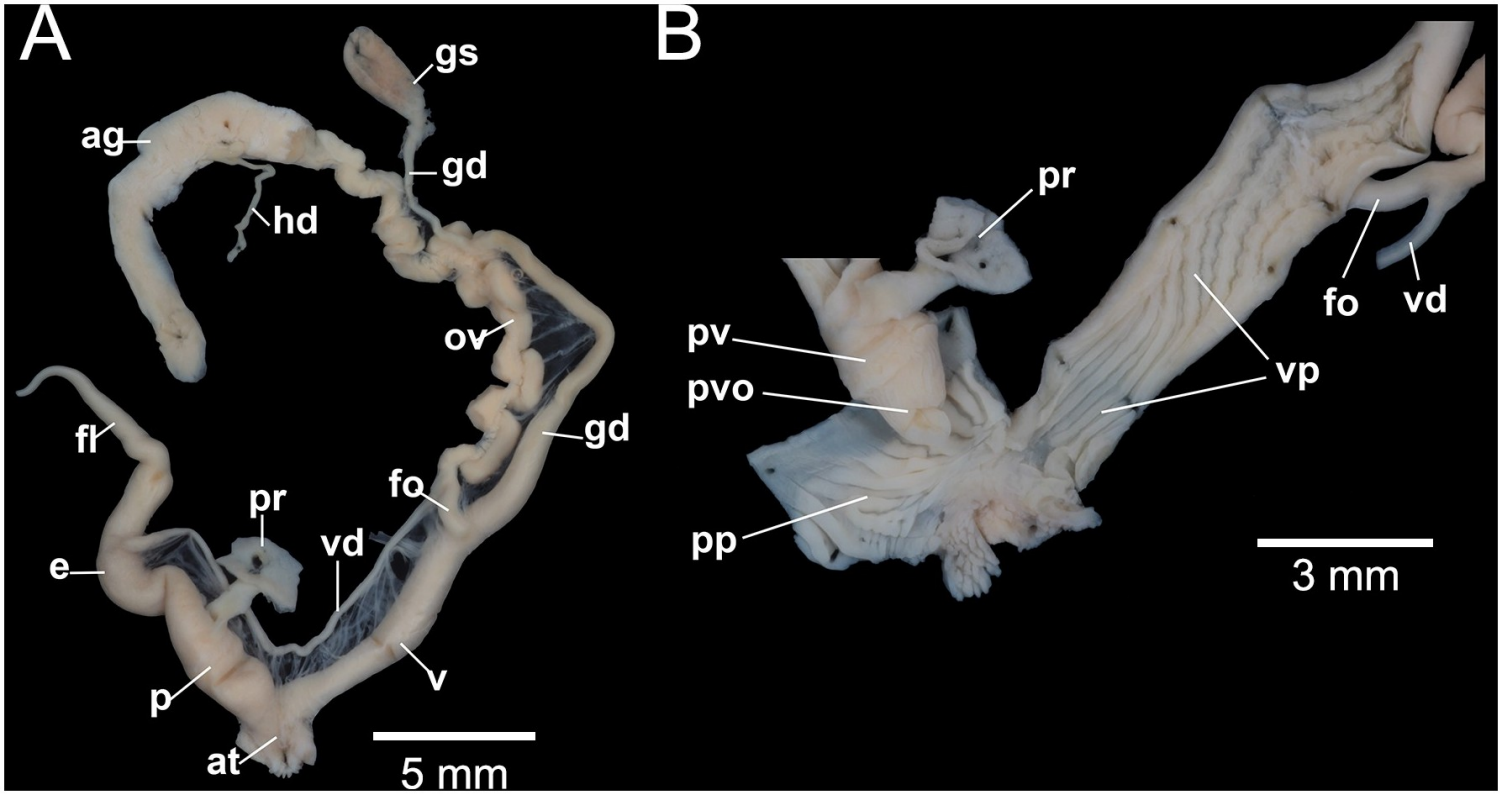
Genital system of *Amphidromus cruentatus* specimen NMNS-8476-058 (ZY6) from Chu Prong, Vietnam. A. General view of genitalia and B. internal wall sculpture of penis and vagina.

The 0.95-probability prediction ellipses of different *A*. *cruentatus* morphs overlap one another in both PCA plots. By combining data of all morphs, the data points of the type specimens of *A*. *eudeli*, *A*. *fuscolabris*, *A*. *thakhekensis*, *A*. *daoae*, *A*. *gerberi bolovenensis*, *A*. *goldbergi*, *A*. *pengzhuoani*, *A*. *eichhorsti* and *A*. *pankowskiae* are well within the 0.95-probability prediction ellipse of *A*. *cruentatus*. However, the data points of the type specimens of *A*. *anhdaoorum*, *A*. *stungtrengensis*, *A*. *yangbayensis* and *A*. *yenlinhae* remain outside the 0.95-probability prediction ellipse of *A*. *cruentatus*. The PCA of all shell parameters also revealed that the ellipse of *A*. *cruentatus* does not significantly overlap with those of *A*. *atricallosus* and *A*. *inversus*. We observed no differences in genitalia or in mean shell shape from the outline-based analysis with respect to site and morph membership of *A*. *cruentatus* (Figs 9 and 10, S1 Fig).

## Discussion

Taxonomy and systematics of the Asian arboreal snail genus *Amphidromus* rely predominantly on conchological characters, such as shell shape and colouration which have been considered particularly informative at the species level [7, 12]. However, extensive intraspecific variation in shell colouration has been reported in some species [9–11]. Uncertainty about the amounts of intraspecific variation in other *Amphidromus* species renders their correct delineation difficult. Species that were described based on rather minor differences in shell features are particularly likely to contribute to taxonomic inflation [13, 21].

The term polymorphism in a wide sense denotes the presence of two or more distinct morphs in a single interbreeding population, determined by genetics, environmental cues, or an interaction between genes and the environment [56]. The study of shell polymorphism in *Amphidromus* has mainly focused on their chirality [31, 33, 57], whereas the polymorphism of shell colouration has been extensively studied in the European land snails *Cepaea* and *Theba* of the family Helicidae [58–61]. This study is thus the first to demonstrate the extent of shell colouration polymorphism in *A*. *cruentatus*. On one hand, the result reveals that both contrasting striped and stripeless morphs from Laos and Vietnam belong to the same mitochondrial lineage. On the other hand, the PCA and shell outline-based analysis indicate an overall similarity in their shell shape, adding to the previous record of genitalia similarity between the two morphs living in sympatry [9]. Thus, the observed differences in shell colouration among the Lao and Vietnamese populations are considered to be well within the range of *A*. *cruentatus* intraspecific variation. The notably high variation in shell colouration within the same genetic lineage has also been reported in the arboreal genus *Aegistohadra* from the same family [33].

The occurrence of shell colouration polymorphism has been explained by several mechanisms such as environment-related frequency differences, background matching, microhabitat distribution, physiological differences between morphs, selective predation and frequency-dependent predation (apostatic selection) [62]. Although shell colouration polymorphism in arboreal snails has been attributed to camouflage from predation [30, 63, 64], other factors such as physiological adaptation to microclimate [65] and differential shell strength [66] could not be disregarded. Further field surveys and experiments are thus needed to elucidate the underlying causes of shell colouration polymorphism in *A*. *cruentatus*.

The examination of type specimens of *A*. *eudeli*, *A*. *fuscolabris*, *A*. *thakhekensis*, *A*. *gerberi bolovenensis*, *A*. *goldbergi*, *A*. *pengzhuoani*, *A*. *eichhorsti* and *A*. *pankowskiae* revealed that these nominal species fall within the intraspecific variation range of *A*. *cruentatus*. *Amphidromus thakhekensis* and *A*. *eichhorsti* correspond to the stripeless morph, whereas the remaining nominal species correspond to the striped morph. Apart from the similarity in shell shape as revealed by PCA, the other characters shared by all these nominal species are their exclusively sinistral shell; the occurrence of pale pink to carmine-purple parietal wall, columella, apical whorls and expanded lip; and a yellow to orange-red subsutural band. Therefore, we agree with some part of synonymizations proposed by Páll-Gergely *et al*. [13] and regard these nominal species as junior synonyms of *A*. *cruentatus* (Table 4).

**Table 4:** Summary of the status of *Amphidromus cruentatus* and similar related species.

Nominal species	Shell morph	Revised taxonomy	Remarks
*A*. *cruentatus* (Morelet, 1875)	stripeless	*A*. *cruentatus*	The oldest nominal taxon among the synonyms of *A*. *cruentatus*.
*A*. *eudeli* Ancey, 1897	striped		Synonyms of *A*. *cruentatus* due to the shell morphometric analyses and the sharing of these shell characters: exclusively sinistral shell; the occurrence of pale pink to carmine-purple parietal wall, columella, apical whorls and expanded lip; and a yellow to orange-red subsutural band. Striped and stripeless morphs from the same collecting locality also belong to the same mitochondrial lineage.
*A*. *fuscolabris* Möllendorff, 1898	striped		
*A*. *thakhekensis* Thach & Huber, 2017	stripeless		
*A*. *gerberi bolovenensis* Thach & Huber, 2018	striped		
*A*. *goldbergi* Thach & Huber, 2018	striped		
*A*. *pengzhuoani* Thach, 2018	striped		
*A*. *eichhorsti* Thach, 2020	stripeless		
*A*. *pankowskiae* Thach, 2020	striped		
*A*. *daoae* Thach, 2016	stripeless	*A*. *daoae*	Distinct from the stripeless morph of *A*. *cruentatus* by a thin and transparent parietal callus, a darker apertural lip, a pinkish columella and a fainter subsutural band, but not distinct from *A*. *cruentatus* in PCA.
*A*. *yangbayensis* Thach & Huber, 2016	striped	*A*. *yangbayensis*	Distinct from the striped morph of *A*. *cruentatus* by a more elongate and slender shell, a thin and transparent parietal callus, a whitish apertural lip, a pinkish columella, and PCA.
*A*. *yenlinhae* Thach & Huber, 2017	striped	*A*. *yenlinhae*	Distinct from the striped morph of *A*. *cruentatus* by a more elongate and slender shell, a thin and transparent parietal callus, a whitish apertural lip, a pinkish columella, a greenish subsutural band, and PCA.
*A*. *anhdaoorum* Thach, 2017	striped	*A*. *anhdaoorum*	Distinct from the striped morph of *A*. *cruentatus* by a thin and transparent parietal callus, a whitish subsutural band, a darker apertural lip and columella, and PCA.
*A*. *stungtrengensis* Thach & Huber, 2018	striped	*A*. *stungtrengensis*	Distinct from the striped morph of *A*. *cruentatus* by a thin and transparent parietal callus, a reddish subsutural band, a darker apertural lip and columella, and PCA.

Pairwise uncorrected interspecific *p*-distances for 16S rRNA among *Amphidromus* species in this study are found to be higher than other genera in the Camaenidae, e.g., *Aegistohadra* from China and Vietnam (5.97–11.86%) [33], *Camaena* from China (5–15%) [35], *Euhadra* (5.8–16.5%) and *Mandarina* (0–10.7%) from Bonin Islands, Japan [67], while comparable to *Acusta* from East Asia (5.3–18.8%) [68]. Although most relationships among *Amphidromus* species still involved an unresolved polytomy, some relationships could be inferred to some extent. Four out of seven *Amphidromus* species groups in the strict sense of Laidlaw and Solem [7], namely *A*. *atricallosus* (including *A*. *leucoxanthus*), *A*. *perversus*, *A*. *martensi* (including *A*. *similis*) and *A*. *palaceus* species groups, belong to the same mitochondrial lineage revealed in this study. Two out of six species groups classified in the subgenus *Syndromus* [7], namely *A*. *xiengensis* (including *A*. *flavus* and *A*. *areolatus*) and *A*. *porcellanus* species groups, constitute the same mitochondrial lineage. Our molecular analyses also retrieved the *A*. *adamsii* species group [7] as a distinct lineage, containing *A*. *adamsii* and *A*. *pictus* from Borneo, and interestingly with the addition of *A*. *principalis* from Kra Island in the Gulf of Thailand, which is 1,700 km far from Borneo.

### Systematic description

**Family Camaenidae Pilsbry**, **1895**

**Genus *Amphidromus* Albers, 1850. Type species**. *Helix perversa* Linnaeus, 1758, by subsequent designation by von Martens [69].

***Amphidromus cruentatus*** (**Morelet**, **1875**)

Figs 2, 3, 4A–4F, 7, 9 and 10

*Bulimus cruentatus* Morelet, 1875: 264, 265, pl. 13, fig. 5. Type locality: Cambodje [Cambodia] [70]. Pfeiffer, 1877: 24, 25 [71].

*Amphidromus cruentatus*—Fischer, 1891: 31 [72]. Fulton, 1896: 89 [73]. Pilsbry, 1900: 187, pl. 60, figs 39, 40 [74]. Fischer and Dautzenberg, 1904: 405 [75]. Laidlaw and Solem, 1961: 524, 614 [7]. Richardson, 1985: 15 [76]. Sutcharit *et al*., 2015: 67, figs 1e, 6f [12]. Páll-Gergely *et al*., 2020: 51, 52 [13].

*Amphidromus eudeli* Ancey, 1897: 63. Type locality: near Binh Dinh, Annam [central Vietnam], in forests [77]. Fischer and Dautzenberg, 1904: 405 [75]. Páll-Gergely *et al*., 2020: 52 [13]. **New synonym**.

*Amphidromus zebrinus fuscolabris* Möllendorff, 1898: 75. Type locality: Boloven [Boloven Plateau, Paksong, Champasak, Laos] [78]. Pilsbry, 1900: 199, 200 [74]. Fischer and Dautzenberg, 1904: 407 [75]. Zilch, 1953: 134, pl. 23, fig. 22 [79]. **New synonym**.

*Amphidromus zebrinus* var. *eudeli*—Pilsbry, 1900: 199, 200, pl. 63, figs 87, 88 [74]. Richardson, 1985: 48 [76].

*Amphidromus* (*Syndromus*) *zebrinus eudeli*—Laidlaw and Solem, 1961: 564, 617 [7].

*Amphidromus* (*Syndromus*) *zebrinus fuscolabris*—Laidlaw and Solem, 1961: 564, 621 [7]. Richardson, 1985: 49 [76].

*Syndromus zebrinus eudeli*—Schileyko, 2011: 52 [80].

*Syndromus zebrinus fuscolabris*—Schileyko, 2011: 52 [80].

*Amphidromus* (*Syndromus*) *fuscolabris*—Inkhavilay *et al*., 2017: 32–34, figs 9e, f, 12g–i, 13j–m, 14c, d [9]. Inkhavilay *et al*., 2019: 89, 90, figs 43a, b, 57g, h [20]. Páll-Gergely *et al*., 2020: 52 [13].

*Amphidromus thakhekensis* Thach & Huber in Thach, 2017: 48, figs 553–556. Type locality: Thakhek, Khammouane, South-Central Laos [14]. Inkhavilay *et al*., 2019: 89, 90 [20]. Páll-Gergely *et al*., 2020: 52, 76 [13]. Thach, 2020: 79, 80 [18].

*Amphidromus gerberi bolovenensis* Thach & Huber in Thach, 2018: 52, 53, figs 663–667. Type locality: Naoh, Attapeu, Boloven Plateau, South Laos [17]. Páll-Gergely *et al*., 2020: 73 [13]. **New synonym**.

*Amphidromus goldbergi* Thach & Huber in Thach, 2018: 53, figs 678–683. Type locality: Saravan [Salavan], South Laos [17]. Páll-Gergely *et al*., 2020: 52, 73 [13].

*Amphidromus pengzhuoani* Thach, 2018: 34, 35, pl. 2, figs 11–13. Type locality: Luang Namtha, Northwest Laos [19]. Páll-Gergely *et al*., 2020: 52, 75 [13].

*Amphidromus eichhorsti* Thach, 2020: 57, 58, figs 660–665. Type locality: North Laos [18]. **New synonym**.

*Amphidromus pankowskiae* Thach, 2020: 72, figs 587–591. Type locality: Northwestern District of Khánh Hòa, Central Vietnam [18]. **New synonym**.

#### Materials examined

Holotype of *Bulimus cruentatus* Morelet, 1875: NHMUK 1893.2.4.163 (Fig 2A). Syntype of *A*. *eudeli* Ancey, 1897: RBINS 617427 (Fig 2C). Holotype of *A*. *zebrinus fuscolabris* Möllendorff, 1898: SMF 7641 (Fig 2B). Holotype of *A*. *thakhekensis* Thach & Huber, 2017: MNHN-IM-2000-33216 (Fig 2D). Holotype of *A*. *gerberi bolovenensis* Thach & Huber, 2018: MNHN-IM-2000-34074 (Fig 2E). Holotype of *A*. *goldbergi* Thach & Huber, 2018: MNHN-IM-2000-34073 (Fig 2F). Holotype of *A*. *pengzhuoani* Thach, 2018: NHMUK 20180243 (Fig 2G). Holotype of *A*. *eichhorsti* Thach, 2020: MNHN-IM-2000-35554 (Fig 2H). Holotype of *A*. *pankowskiae* Thach, 2020: MNHN-IM-2000-35543 (Fig 2I). Samphanh, Phongsali, Laos: NMNS-8476-001 to NMNS-8476-051 (stripeless morph: 10 shells and 8 specimens in ethanol; Fig 3A–3C; striped morph: 24 shells and 9 specimens in ethanol; Fig 3D–3F). Chu Prong, Gia Lai, Vietnam: NMNS-8476-052 to NMNS-8476-066 (striped morph: 5 shells and 10 specimens in ethanol; Fig 3G–3I). Ban Phone, La-Marm, Sekong, Laos: CUMZ 7040, 7042 (stripeless morph: 83 shells; Inkhavilay et al, 2017: 17, fig. 13l, m [9], Fig 4A and 4B; striped morph: 34 shells; Inkhavilay et al, 2017: 17, fig. 13j, k [9]; Fig 4C and 4D). Ban Xai Na Pho, Phatumphone, Champasak, Laos: CUMZ 7044 (stripeless morph: 1 shell; Fig 4E; striped morph: 1 shell; Fig 4F).

#### Diagnosis

Peristome pale pink to carmine-purple on the broadly expanded lip, columella, and parietal wall; roseate to brownish tint on the apical two whorls; subsutural bands yellow to orange-red; monochrome yellow shell or with green to brown-black axial stripes.

#### Measurements

Shell height: range 22.0–45.0 mm and average 33.5 ± 4.6 mm. Shell width: range 12.1–22.4 mm and average 16.9 ± 1.9 mm.

#### Description

Shell medium, rather thin and glossy, elongated-conical, monomorphic sinistral. Spire conical with nearly smooth surface; suture wide and shallow. Apex acute, without black spot; following two whorls roseate to brownish tint. Whorls 6 to 7 with little convex whorls. Periostracum thin corneous and transparent. Shell background white to yellow; shell colouration varying from monochrome yellow or green (stripeless morph) to with variegated brown to dark-green slanted blotches or axial stripes (striped morph); subsutural band always present with yellow to orange-red color. Last whorl large, rounded, sometimes sub-peripheral bands partially present; varix wanting. Parietal callus thick or thin, stained with pale pink to carmine-purple. Aperture elongated auriform and angulated below; peristome slightly thickened, expanded and not reflected. Lip stained with white or pale pink to carmine-purple; inside aperture pale pink to carmine-purple. Columella somewhat thickened, straight, dilated margin, pale pink to carmine-purple. Umbilicus imperforate; umbilical area pale yellow to orange (Figs 2, 3 and 4A–4F).

Shell variation. There are two different major patterns in *A*. *cruentatus*: (a) stripeless morph: monochrome yellow without stripes (Figs 2A, 2D and 2H, 3A–3C, 4A and 4B and 5E), and (b) striped morph: with green to brown-black axial slanted streaks or merged blotches on teleoconch (Figs 2B, 2C, 2E–2G and 2I, 3D–3I and 4C, 4D and 4F). The striped morph is also different from the stripeless morph in having mild to moderate roseate to brownish tint on the apical two whorls, and distinct reddish-brown dots on the second and third whorls.

#### Genitalia

Correspond to the genitalia description of *A*. *fuscolabris* in Inkhavilay *et al*. (2017: fig. 14c, d) [9] (Fig 10).

#### Distribution

1. Stripeless morph only: Cambodia [70]; Thakhek, Khammouane, Laos [14]; North Laos [18].
2. Striped morph only: Binh Dinh, Vietnam [75, 77]; Paksong, Champasak, Laos [78]; Attapeu, Laos; Salavan, Laos [17]; Luang Namtha, Northwest Laos [19]; Khanh Hoa, Vietnam [18]; Chu Prong, Gia Lai, Vietnam.
3. Both morphs coexist: Ban Phone, La-Marm, Sekong, and Ban Xai Na Pho, Phatumphone, Champasak, Laos [9]; Samphanh, Phongsali, Laos (Fig 1).

#### Remarks

Five nominal species were previously treated as junior synonyms of *A*. *cruentatus* [13], but they are retained as valid in this study based on some shell characters and PCA (Table 4). Although the data point representing the *A*. *daoae* holotype is well within the 0.95-probability prediction ellipse of *A*. *cruentatus* in both PCA, we decide to retain *A*. *daoae* and its subspecies as distinct from *A*. *cruentatus* based on shell characters, in contrast to Páll-Gergely *et al*.’s treatment as junior synonyms [13]. *Amphidromus daoae* (Fig 4G) and *A*. *daoae robertabbasi* are slightly different from the stripeless morph of *A*. *cruentatus* by having a thin and transparent parietal callus, a darker apertural lip, a pinkish columella and a fainter subsutural band [16], while not having the pale pink to carmine-purple parietal wall and apical whorls, and a yellow to orange-red subsutural band as in *A*. *cruentatus*. Both PCA and shell characters suggest that *A*. *anhdaoorum*, *A*. *stungtrengensis*, *A*. *yangbayensis* and *A*. *yenlinhae* are distinct from *A*. *cruentatus*. This is in contrast to Páll-Gergely *et al*.’s treatments of *A*. *anhdaoorum* and *A*. *stungtrengensis* as junior synonyms of *A*. *fuscolabris*, and *A*. *yangbayensis* and *A*. *yenlinhae* as junior synonyms of *A*. *eudeli* [13]. *Amphidromus yangbayensis* and *A*. *yenlinhae* are very similar, but they differ from the striped morph of *A*. *cruentatus* by having a more elongate and slender shell, a thin and transparent parietal callus, a whitish apertural lip, a pinkish columella, and an additional greenish subsutural band in *A*. *yenlinhae* (Fig 4H and 4I). This distinction from the striped morph of *A*. *cruentatus* was previously pointed out in the case of *A*. *yenlinhae* [22]. Furthermore, *A*. *stungtrengensis* differs from the striped morph of *A*. *cruentatus* by having a thin and transparent parietal callus, a reddish subsutural band, and a darker apertural lip and columella (Fig 4J). *Amphidromus anhdaoorum* also differs by having a thin and transparent parietal callus, a whitish subsutural band, and a darker apertural lip and columella (Fig 4K). Although *A*. *yangbayensis* and *A*. *yenlinhae* are very similar in shell shape and colouration, the consideration of their species’ distinction is beyond the scope of this study.

## Supporting information

S1 Fig Genital system of *Amphidromus cruentatus*. A., B. specimens NMNS-8476-009 (X79) and C., D. NMNS-8476-034 (X81) from Samphanh, Phongsali, Laos showing A., C. general view of genitalia and B., D. internal wall sculpture of penis and vagina.
(TIF)

S1 Table List of *Amphidromus* and outgroup species used in molecular phylogenetic analyses with their locality details and accession numbers. (PDF)

S2 Table Percentage of uncorrected pairwise interspecific distances (below the diagonal) and intraspecific distances (diagonal) for the partial 16S rRNA gene fragments among different *Amphidromus* species (different subspecific taxa were combined together). (PDF)

S3 Table Shell parameters of *Amphidromus cruentatus* specimens. (PDF)

## Abbreviations

ag: albumen gland
at: atrium
D: dextral
e: epiphallus
fl: flagellum
fo: free oviduct
gd: gametolytic duct
gs: gametolytic sac
hd: hermaphroditic duct
ov: oviduct
p: penis
pp: penial pilaster
pr: penial retractor muscle
pv: penial verge
pvo: penial verge orifice
S: sinistral
v: vagina
vd: vas deferens
vp: vaginal pilaster
CUMZ: Chulalongkorn University Museum of Zoology, Bangkok
MNHN: Muséum national d’Histoire naturelle, Paris
NHM: The Natural History Museum, London
NHMUK: when citing specimen lots deposited in NHM
NMNS: National Museum of Natural Science of Taiwan, Taichung
RBINS: Royal Belgian Institute of Natural Sciences, Brussels
RMNH: Naturalis Biodiversity Center, Zoology Collections (Rijksmuseum van Natuurlijke Historie), Leiden
SMF: Senckenberg Forschungsinstitut und Naturmuseum, Frankfurt am Main
