# Supplementary figures and images for: Arboreal snail genus Amphidromus Albers, 1850 of Southeast Asia: Shell polymorphism of Amphidromus cruentatus (Morelet, 1875) revealed by phylogenetic and morphometric analyses
Source: PLoS One. 2022 Aug 29;17(8):e0272966. doi: 10.1371/journal.pone.0272966 (PMC9423684; doi:10.1371/journal.pone.0272966)

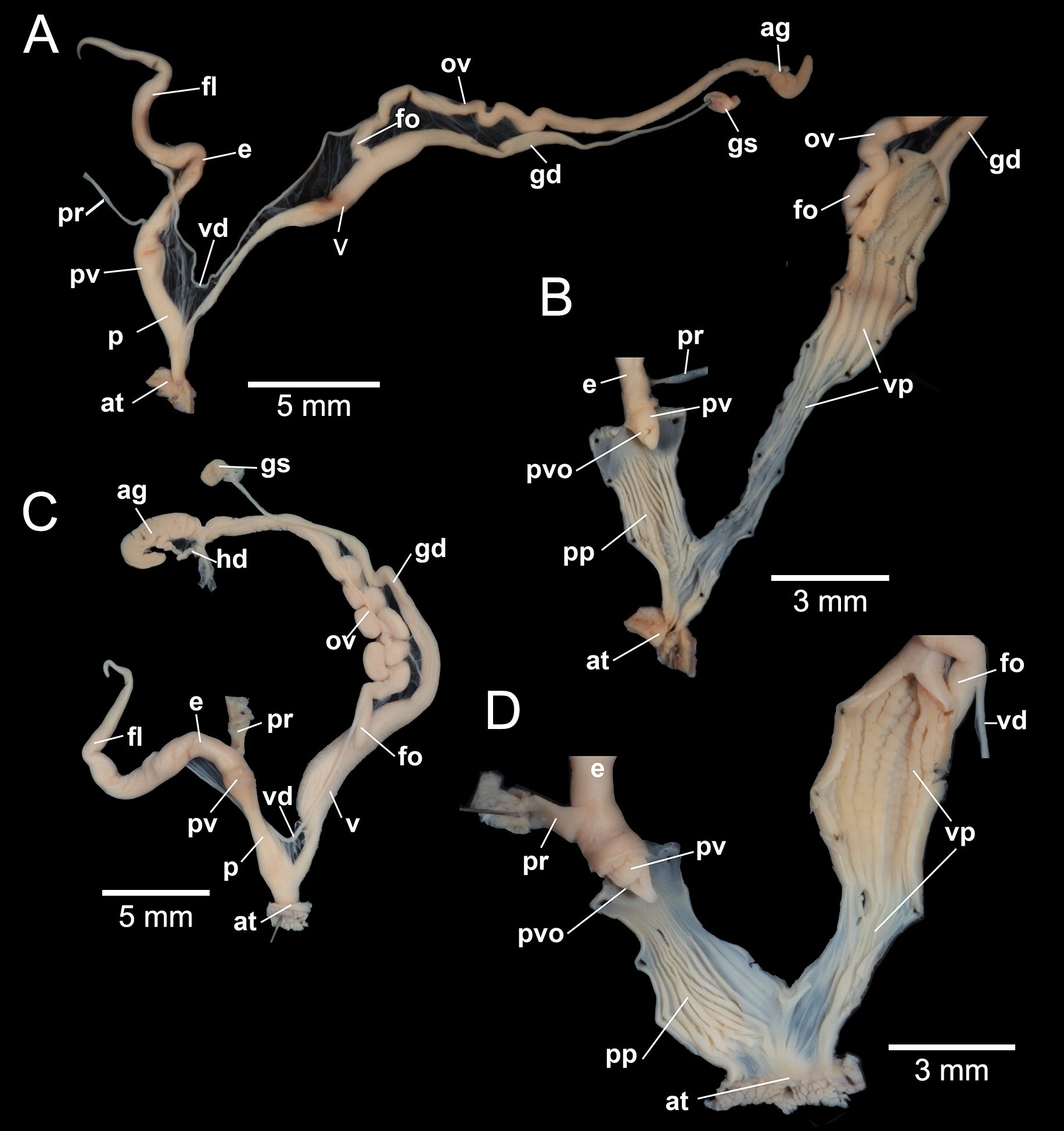

Supplement: S1 Fig — A., B. specimens NMNS-8476-009 (X79) and C., D. NMNS-8476-034 (X81) from Samphanh, Phongsali, Laos showing A., C. general view of genitalia and B., D. internal wall sculpture of penis and vagina. (TIF) [file pone.0272966.s001.tif]
