# Supplementary material for: Arboreal snail genus Amphidromus Albers, 1850 of Southeast Asia: Shell polymorphism of Amphidromus cruentatus (Morelet, 1875) revealed by phylogenetic and morphometric analyses
Source: PLoS One. 2022 Aug 29;17(8):e0272966. doi: 10.1371/journal.pone.0272966 (PMC9423684; doi:10.1371/journal.pone.0272966)
Supplement: S1 Table — (PDF) [file pone.0272966.s002.pdf]

**S1 Table.**

| No. | Species | Locality | Specimen code / voucher number | Accession numbers | | References |
|---|---|---|---|---|---|---|
| | | | | COI | 16S rDNA | |
| 1 | *Amphidromus adamsii* (Reeve, 1848) | Banggi, Sabah, Malaysia | - | - | AB112370 | [1] |
| 2 | *Amphidromus areolatus* (Pfeiffer, 1861) | Muang, Pang Nga, Thailand | - | - | AB112387 | |
| | | Khao Poo-Khao Ya National Park, Patthalung, Thailand | - | - | AB112405 | |
| 3 | *Amphidromus atricallosus atricallosus* (Gould, 1843) | Hot Spring, Muang, Ranong, Thailand | - | - | AB112365 | |
| | | Ban Takun, Suratthani, Thailand | - | - | AB112393 | |
| | | Muang, Pangnga, Thailand | - | - | AB112394 | |
| | | 15 km E of Hangapru, 5 km S of logging camp, Tanintharyi, Myanmar | FLMNH494159 | MF983563 | - | [2] |
| | | 22 km E of Hankadin, 20 km S of logging camp, Yau Hay Huu, Tanintharyi, Myanmar | FLMNH494162 | MF983564 | - | [3] |
| | | 15 km ENE of Hangapru, 2 km S of logging camp, Tanintharyi, Myanmar | FLMNH494103 | MF983565 | - | [4] |
| 4 | *Amphidromus atricallosus temasek* Tan, Chan & Panha, 2011 | Nee Soon, Singapore | | - | AB112368 | [1] |
| 5 | *Amphidromus contrarius* (Müller, 1774) | - | AM | KP085343 | KP085033 | [5] |
| | | 6.5 km N of Los Palos, Lautem District, Timor-Leste | AM_C.468733 | KP085341 | KP085031 | |
| | | south east coast, Beaco, Viqueque District, Timor-Leste | AM_C.468737 | KP085342 | KP085032 | |
| | | north east coast, Com, ca 0.5 km SE of the habour, Lautem District, Timor-Leste | AM_C.468738 | KP085345 | KP085035 | |
| | | Loihuno, Viqueque District, Timor-Leste | AM_C.468774 | KP085344 | KP085034 | |
| 6 | *Amphidromus flavus* (Pfeiffer, 1861) | Luang Pra Bang, Laos | - | - | AB112386 | [1] |
| 7 | *Amphidromus givenchyi* Geret, 1912 | Phu Lom Khao, Na Ku, Kalasin, Thailand | - | - | AB112372 | |
| | | Savanakhet, Laos | - | - | AB112398 | |
| | | Phataem National Park, Ubonratchathani, Thailand | - | - | AB112399 | |

| No. | Species | Locality | Specimen code / voucher number | Accession numbers | | References |
|---|---|---|---|---|---|---|
| | | | | COI | 16S rDNA | |
| 8 | *Amphidromus glaucolarynx* (Dohrn, 1861) | Pong Phu Ron, Thong Pha Phum National Park, Kanchanaburi, Thailand | | - | AB112382 | |
| | | Sai Yok Noi Waterfall, Sai Yok, Kanchanaburi, Thailand | | - | AB112383 | |
| | | Kaeng Kracharn National Park, Petchaburi, Thailand | | - | AB112384 | |
| | | Pra Kra Yang Cave, Kraburi, Ranong, Thailand | | - | AB112385 | |
| 9 | *Amphidromus inversus annamiticus* (Crosse & Fischer, 1863) | Elar Island, Sattahip, Chonburi, Thailand | | - | AB112366 | |
| | | Na Muang Waterfall, Koh Samui, Suratthani, Thailand | | - | AB112391 | |
| 10 | *Amphidromus inversus inversus* (Müller, 1774) | Kapas Island, Malaysia | | FJ472655 | - | [6] |
| | | Botanical Garden, Singapore | | | AB112367 | [1] |
| | | Kabupaten Lampung Selatan, Indonesia | | | AB112400 | |
| 11 | *Amphidromus leucoxanthus* (Martens, 1864) | Khao Ang Rue Nai Wildlife Sanctuary, Chachoengsao, Thailand | - | - | AB112369 | |
| | | Makok Waterfall, Plieu National Park, Chanthaburi, Thailand | - | - | AB112392 | |
| | | Tioman Island, Malaysia | - | - | AB112395 | |
| 12 | *Amphidromus martensi* Boettger, 1893 | Danum Valley, Sabah, Malaysia | - | - | AB112376 | |
| 13 | *Amphidromus palaceus* (Mousson, 1849) | Pangandaran, Indonesia | - | - | AB112374 | |
| 14 | *Amphidromus perversus natunensis* Fulton, 1906 | Natuna Island, Indonesia | - | - | AB112375 | |
| 15 | *Amphidromus perversus perversus* (Linnaeus, 1758) | Bali Island, Indonesia | AM19 / CUMZ 4291 | MW649970 | MW652850 | [7] |
| 16 | *Amphidromus pictus* Fulton, 1896 | Sukau, Sabah, Malaysia | - | - | AB112381 | [1] |
| 17 | *Amphidromus porcellanus* (Mousson, 1849) | Bogor, Indonesia | - | - | AB112380 | |
| 18 | *Amphidromus principalis* Sutcharit & Panha, 2015 | Kra Island, Pak Panang, Nakhon Sri Thammarat, Thailand | - | - | AB112378 | |

| No. | Species | Locality | Specimen code / voucher number | Accession numbers | | References |
|---|---|---|---|---|---|---|
| | | | | COI | 16S rDNA | |
| 19 | *Amphidromus schomburgki dextrochlorus* Sutcharit & Panha, 2006 | Ban Khok klang, Tao Ngoi, Sakonnakhon, Thailand | - | - | AB112373 | |
| 20 | *Amphidromus schomburgki schomburgki* (Pfeiffer, 1860) | Kud Island, Koh Kud, Trat, Thailand | - | - | AB112396 | |
| 21 | *Amphidromus semitessellatus* (Morlet, 1885) | Khao Ang Rue Nai Wildlife Sanctuary, Chachoengsao, Thailand | - | - | AB112379 | |
| | | Makok Waterfall, Plieu National Park, Chanthaburi, Thailand | - | - | AB112403 | |
| | | Trong Nong Waterfall, Plieu National Park, Chanthaburi, Thailand | - | - | AB112404 | |
| 22 | *Amphidromus similis* Pilsbry, 1900 | Sarawak, Malaysia | - | - | AB112371 | |
| 23 | *Amphidromus xiengensis* Morlet, 1891 | Mae Yom National Park, Phrae, Thailand | - | - | AB112377 | |
| | | Tam Chieng Dao, Chieng Dao, Chiang Mai, Thailand | - | - | AB112397 | |
| | | Elar Island, Sattahip, Chonburi, Thailand | - | - | AB112401 | |
| | | Ban Takun, Suratthani, Thailand | - | - | AB112402 | |
| 24 | *Amphidromus* sp. BSH-2002 | Southern Thailand | - | AY148562 | - | [8] |
| 25 | *Camaena cicatricosa* (Müller, 1774) | Guiping, Guangxi, China | FJIQBC18503 | KU061276 | KU586474 | [9] |
| 26 | *Camaena poyuensis* Zhou, Wang & Ding, 2016 | Poyue town, Bama, Hechi, Guangxi, China | FJIQBC18484 | KU061273 | KU586468 | |
| 27 | *Trichelix hiraseana* (Pilsbry, 1905) | Taiwan | 3901 | EF204842 | EF204805 | [10] |
| 28 | *Cornu aspersum* (Müller, 1774) (Helicidae) | Italy | 133 | KU586502 | KU586459 | [9] |

**References**

1. Sutcharit C, Asami T, Panha S. Evolution of whole-body enantiomorphy in the tree snail genus *Amphidromus*. J Evol Biol. 2007;20:661–72. doi: https://doi.org/10.1111/j.1420-9101.2006.01246.x.

2. Slapcinsky J, Mulcahy D. Accession No. MF983563, *Amphidromus atricallosus,* voucher FLMNH494159, cytochrome c oxidase subunit I (COI) gene; [cited 5 October 2021]. Available from: https://www.ncbi.nlm.nih.gov/nuccore/MF983563. 2017.

3. Slapcinsky J, Mulcahy D. Accession No. MF983564, *Amphidromus atricallosus*, voucher FLMNH494162, cytochrome c oxidase subunit I (COI) gene; [cited 5 October 2021]. Available from: https://www.ncbi.nlm.nih.gov/nuccore/MF983564. 2017.

4. Slapcinsky J, Mulcahy D. Accession No. MF983565, *Amphidromus atricallosus*, voucher FLMNH494103, cytochrome c oxidase subunit I (COI) gene; [cited 5 October 2021]. Available from: https://www.ncbi.nlm.nih.gov/nuccore/MF983565. 2017.

5. Köhler F, Criscione F. A molecular phylogeny of camaenid land snails from north-western Australia unravels widespread homoplasy in morphological characters (Gastropoda, Helicoidea). Mol Phylogen Evol. 2015;83:44–55. doi: https://doi.org/10.1016/j.ympev.2014.11.009.

6. Schilthuizen M, Looijestijn S. The sexology of the chirally dimorphic snail species *Amphidromus inversus* (Gastropoda: Camaenidae). Malacologia. 2009;51(2):379−87.

7. Jirapatrasilp P, Huang C-W, Hwang C-C, Sutcharit C, Lee C-T. Convergent evolution of *Amphidromus*-like colourful arboreal snails and phylogenetic relationship of East Asian camaenids, with description of a new *Aegistohadra* species (Helicoidei, Camaenidae, Bradybaeninae). Invertebr Syst. 2022;36(3):244–90.

8. Rundell RJ, Holland BS, Cowie RH. Accession No. AY148562, *Amphidromus* sp., voucher BSH-2002, cytochrome c oxidase subunit I (COI) gene; [cited 29 October 2021]. Available from: https://www.ncbi.nlm.nih.gov/nuccore/ay148562. 2002.

9. Ding H-L, Wang P, Qian Z-X, Lin J-H, Zhou W, Hwang C, et al. Revision of sinistral land snails of the genus *Camaena* (Stylommatophora, Camaenidae) from China based on morphological and molecular data, with description of a new species from Guangxi, China. ZooKeys. 2016;584:25–48.

10. Wu S-P, Hwang C-C, Lin Y-S. Systematic revision of the arboreal snail *Satsuma albida* species complex (Mollusca: Camaenidae) with descriptions of 14 new species from Taiwan. Zool J Linn Soc. 2008;154(3):437–93. doi: https://doi.org/10.1111/j.1096-3642.2008.00415.x.
