# Supplementary material for: Arboreal snail genus Amphidromus Albers, 1850 of Southeast Asia: Shell polymorphism of Amphidromus cruentatus (Morelet, 1875) revealed by phylogenetic and morphometric analyses
Source: PLoS One. 2022 Aug 29;17(8):e0272966. doi: 10.1371/journal.pone.0272966 (PMC9423684; doi:10.1371/journal.pone.0272966)
Supplement: S2 Table — (PDF) [file pone.0272966.s003.pdf]

S2 Table.

Taxa	**1.**	2.	3.	4.	5.	6.	7.	8.	9.	10.	11.	12.	13.	14.	15.	16.	17.	18.	19.	20.
**1. *cruentatus***	1.23																			
2. *adamsii*	15.53	-																		
3. *areolatus*	17.60	13.79	10.92																	
4. *atricallosus*	17.76	17.74	18.21	4.02																
5. *contrarius*	17.90	11.09	11.90	17.79	6.44															
6. *flavus*	16.64	11.59	6.81	16.16	11.83	-														
7. *givenchyi*	14.78	18.30	19.11	12.98	19.23	16.91	0.77													
8. *glaucolarynx*	16.48	18.32	18.53	15.19	19.21	17.32	16.31	3.54												
9. *inversus*	13.77	15.23	16.67	13.02	16.22	15.43	11.40	16.09	2.25											
10. *leucoxanthus*	16.72	16.95	17.91	6.82	17.05	16.52	14.75	16.26	14.25	3.26										
11. *martensi*	16.76	16.38	17.39	6.68	17.07	16.23	13.03	14.15	13.00	8.43	-									
12. *palaceus*	15.03	17.24	16.24	6.11	16.90	15.65	13.03	16.16	13.29	6.99	8.05	-								
13. *perversus*	15.68	15.69	15.76	4.29	15.57	13.48	13.32	14.13	11.99	6.10	6.39	4.80	1.47							
14. *pictus*	16.02	5.46	14.66	17.74	12.53	13.91	18.58	17.60	16.38	17.05	15.52	16.67	15.40	-						
15. *porcellanus*	17.42	13.62	9.13	17.25	14.09	5.85	17.58	17.83	16.16	17.87	15.65	17.39	15.82	14.20	-					
16. *principalis*	15.14	7.18	12.79	17.74	12.18	13.04	18.87	18.39	17.10	15.90	15.23	16.09	15.98	6.32	13.33	-				
17. *schomburgki*	17.92	19.11	18.46	15.16	18.82	17.97	13.51	17.74	12.21	16.57	15.09	14.94	13.80	17.10	18.55	17.53	1.72			
18. *semitessalatus*	17.98	14.11	8.06	19.10	11.84	10.45	19.35	19.58	17.11	18.84	17.56	17.37	16.40	13.73	12.00	11.81	18.28	9.80		
19. *similis*	17.87	17.87	18.16	6.77	18.10	16.57	14.51	14.99	13.98	7.30	6.63	8.65	6.41	17.87	16.28	16.71	13.54	18.48	-	
20. *xiengensis*	16.60	12.79	7.97	17.65	12.50	4.06	17.62	18.25	16.07	17.74	16.81	15.80	14.96	14.51	7.17	13.36	18.39	11.13	17.94	5.80
