# Supplementary material for: Arboreal snail genus Amphidromus Albers, 1850 of Southeast Asia: Shell polymorphism of Amphidromus cruentatus (Morelet, 1875) revealed by phylogenetic and morphometric analyses
Source: PLoS One. 2022 Aug 29;17(8):e0272966. doi: 10.1371/journal.pone.0272966 (PMC9423684; doi:10.1371/journal.pone.0272966)
Supplement: S3 Table — (PDF) [file pone.0272966.s004.pdf]

S3 Table.

Shell measurements (mm)	Samphanh, Phongsali, Laos			Ban Phone, La-Marm, Sekong, Laos			Ban Xai Na Pho, Phatumphone, Champasak, Laos			Chu Prong, Gia Lai, Vietnam	Total (N = 108)
	stripeless morph (N = 18)	striped morph (N = 33)	Total (N = 51)	stripeless morph (N = 20)	striped morph (N = 20)	Total (N = 40)	stripeless morph (N = 1)	striped morph (N = 1)	Total (N = 2)	striped morph (N = 15)	
H	33.05 ± 2.91	31.74 ± 3.17	32.20 ± 3.11	37.74 ± 2.79	37.71 ± 3.04	37.72 ± 2.88	39.8	36.8	38.30	27.14 ± 2.79	33.66 ± 4.71
D	16.46 ± 0.88	16.33 ± 1.20	16.38 ± 1.09	18.61 ± 1.25	18.81 ± 1.28	18.71 ± 1.26	19.29	18.91	19.10	14.25 ± 1.26	16.99 ± 1.94
AH	14.87 ± 1.18	14.55 ± 1.33	14.66 ± 1.28	17.29 ± 1.15	17.55 ± 1.23	17.42 ± 1.18	18.26	17.13	17.70	12.40 ± 1.27	15.43 ± 2.15
AW	9.34 ± 0.60	9.38 ± 0.78	9.37 ± 0.71	10.08 ± 0.79	10.21 ± 0.69	10.14 ± 0.73	10.30	9.91	10.11	8.32 ± 0.79	9.52 ± 0.94
LWH	22.76 ± 1.79	21.95 ± 2.06	22.23 ± 1.99	26.07 ± 1.86	26.05 ± 1.70	26.06 ± 1.76	26.53	25.41	25.97	18.78 ± 1.64	23.24 ± 3.11
PW	14.01 ± 0.79	13.64 ± 0.84	13.77 ± 0.83	15.42 ± 1.02	15.36 ± 0.90	15.39 ± 0.95	16.08	14.72	15.40	12.06 ± 0.82	14.16 ± 1.43
H-LWH	10.29 ± 1.30	9.79 ± 1.24	9.97 ± 1.27	11.67 ± 1.04	11.66 ± 1.45	11.66 ± 1.24	13.27	11.39	12.33	8.36 ± 1.26	10.42 ± 1.70
SpH	18.18 ± 1.86	17.19 ± 2.01	17.54 ± 2.00	20.45 ± 1.85	20.15 ± 2.16	20.30 ± 1.99	21.54	19.67	20.61	14.74 ± 1.69	18.23 ± 2.71
H/D	2.01 ± 0.10	1.94 ± 0.08	1.96 ± 0.09	2.03 ± 0.08	2.00 ± 0.08	2.02 ± 0.08	2.06	1.95	2.00	1.90 ± 0.06	1.98 ± 0.09
AH/AW	1.59 ± 0.05	1.55 ± 0.06	1.57 ± 0.06	1.72 ± 0.05	1.72 ± 0.04	1.72 ± 0.05	1.77	1.73	1.75	1.49 ± 0.05	1.61 ± 0.10
H/AH	2.22 ± 0.07	2.18 ± 0.08	2.20 ± 0.08	2.18 ± 0.08	2.15 ± 0.10	2.17 ± 0.09	2.18	2.15	2.16	2.19 ± 0.09	2.18 ± 0.09
D/AW	1.76 ± 0.05	1.74 ± 0.06	1.75 ± 0.06	1.85 ± 0.04	1.84 ± 0.06	1.85 ± 0.05	1.87	1.91	1.89	1.71 ± 0.04	1.78 ± 0.07
H/LWH	1.45 ± 0.04	1.45 ± 0.03	1.45 ± 0.03	1.45 ± 0.02	1.45 ± 0.03	1.45 ± 0.03	1.50	1.45	1.47	1.44 ± 0.04	1.45 ± 0.03
LWH/(H-LWH)	2.23 ± 0.18	2.25 ± 0.16	2.24 ± 0.16	2.24 ± 0.11	2.25 ± 0.18	2.25 ± 0.15	2.00	2.23	2.12	2.27 ± 0.22	2.25 ± 0.17
LWH/AH	1.53 ± 0.03	1.51 ± 0.04	1.52 ± 0.04	1.51 ± 0.03	1.49 ± 0.05	1.50 ± 0.04	1.45	1.48	1.47	1.52 ± 0.04	1.51 ± 0.04
SpH/AH	1.22 ± 0.07	1.18 ± 0.08	1.20 ± 0.08	1.18 ± 0.08	1.15 ± 0.10	1.17 ± 0.09	1.18	1.15	1.16	1.19 ± 0.09	1.18 ± 0.09
D/PW	1.17 ± 0.03	1.20 ± 0.03	1.19 ± 0.04	1.21 ± 0.02	1.22 ± 0.03	1.22 ± 0.03	1.20	1.28	1.24	1.18 ± 0.04	1.20 ± 0.04
PW/AW	1.50 ± 0.04	1.46 ± 0.06	1.47 ± 0.06	1.53 ± 0.04	1.51 ± 0.07	1.52 ± 0.05	1.56	1.49	1.52	1.45 ± 0.06	1.49 ± 0.06

H: shell height, D: shell width, AH: apertural height, AW: apertural width, LWH: last whorl height, PW: penultimate whorl width, H-LWH: shell height other than last whorl, SpH: spire height.
